# Two high-quality *de novo* genomes from single ethanol-preserved specimens of tiny metazoans (Collembola)

**DOI:** 10.1093/gigascience/giab035

**Published:** 2021-05-21

**Authors:** Clément Schneider, Christian Woehle, Carola Greve, Cyrille A D'Haese, Magnus Wolf, Michael Hiller, Axel Janke, Miklós Bálint, Bruno Huettel

**Affiliations:** LOEWE Centre for Translational Biodiversity Genomics (LOEWE-TBG), Senckenberganlage 25, 60325 Frankfurt am Main, Germany; Senckenberg Gesellschaft für Naturforschung, Abteilung Bodenzoologie, Am Museum 1, 02826 Görlitz, Germany; Max Planck Institute for Plant Breeding Research, Max Planck Genome-centre Cologne, Carl-von-Linné-Weg 10, 50829 Cologne, Germany; LOEWE Centre for Translational Biodiversity Genomics (LOEWE-TBG), Senckenberganlage 25, 60325 Frankfurt am Main, Germany; Unité Mécanismes adaptatifs & Evolution (MECADEV), CNRS, Muséum national d'Histoire naturelle, 45 rue Buffon 75005 Paris, France; LOEWE Centre for Translational Biodiversity Genomics (LOEWE-TBG), Senckenberganlage 25, 60325 Frankfurt am Main, Germany; Senckenberg Biodiversity and Climate Research Centre, Senckenberganlage 25, 60325 Frankfurt am Main, Germany; Goethe University, Max-von-Laue-Str. 9, 60438 Frankfurt am Main, Germany; LOEWE Centre for Translational Biodiversity Genomics (LOEWE-TBG), Senckenberganlage 25, 60325 Frankfurt am Main, Germany; Goethe University, Max-von-Laue-Str. 9, 60438 Frankfurt am Main, Germany; Senckenberg Research Institute, Senckenberganlage 25, 60325 Frankfurt, Germany; LOEWE Centre for Translational Biodiversity Genomics (LOEWE-TBG), Senckenberganlage 25, 60325 Frankfurt am Main, Germany; Senckenberg Biodiversity and Climate Research Centre, Senckenberganlage 25, 60325 Frankfurt am Main, Germany; Goethe University, Max-von-Laue-Str. 9, 60438 Frankfurt am Main, Germany; LOEWE Centre for Translational Biodiversity Genomics (LOEWE-TBG), Senckenberganlage 25, 60325 Frankfurt am Main, Germany; Senckenberg Biodiversity and Climate Research Centre, Senckenberganlage 25, 60325 Frankfurt am Main, Germany; Max Planck Institute for Plant Breeding Research, Max Planck Genome-centre Cologne, Carl-von-Linné-Weg 10, 50829 Cologne, Germany

**Keywords:** long-read genome sequencing, PacBio, soil invertebrates, eukaryote biodiversity, low-input DNA, integrative taxonomy

## Abstract

**Background:**

Genome sequencing of all known eukaryotes on Earth promises unprecedented advances in biological sciences and in biodiversity-related applied fields such as environmental management and natural product research. Advances in long-read DNA sequencing make it feasible to generate high-quality genomes for many non–genetic model species. However, long-read sequencing today relies on sizable quantities of high-quality, high molecular weight DNA, which is mostly obtained from fresh tissues. This is a challenge for biodiversity genomics of most metazoan species, which are tiny and need to be preserved immediately after collection. Here we present *de novo* genomes of 2 species of submillimeter Collembola. For each, we prepared the sequencing library from high molecular weight DNA extracted from a single specimen and using a novel ultra-low input protocol from Pacific Biosciences. This protocol requires a DNA input of only 5 ng, permitted by a whole-genome amplification step.

**Results:**

The 2 assembled genomes have N50 values >5.5 and 8.5 Mb, respectively, and both contain ∼96% of BUSCO genes. Thus, they are highly contiguous and complete. The genomes are supported by an integrative taxonomy approach including placement in a genome-based phylogeny of Collembola and designation of a neotype for 1 of the species. Higher heterozygosity values are recorded in the more mobile species. Both species are devoid of the biosynthetic pathway for β-lactam antibiotics known in several Collembola, confirming the tight correlation of antibiotic synthesis with the species way of life.

**Conclusions:**

It is now possible to generate high-quality genomes from single specimens of minute, field-preserved metazoans, exceeding the minimum contig N50 (1 Mb) required by the Earth BioGenome Project.

## Introduction

Biodiversity genomics uses genome-scale data to study the molecular basis of biodiversity. New genome data and their analyses are currently revolutionizing life sciences and environmental sciences by addressing scientific questions on evolution, phylogeny, ecology, medicine, and other fields of life sciences. One year after the start of the LOEWE Center for Translational Biodiversity Genome (LOEWE-TBG), the Earth BioGenome Project (EBP) announced plans to sequence reference genomes from all known ∼1.5 M eukaryotic species [1]. High-quality (highly contiguous and complete, preferentially chromosome-level) genomes sequenced from accurately species-identified organisms are essential for these efforts. To achieve its goal, the biodiversity genomics faces a major challenge: most of the eukaryotic biodiversity belongs to highly diverse families of tiny species [2] that are (i) difficult to sequence and (ii) difficult to identify.

Advances in long-read sequencing technology changed the game for biodiversity genomics because this technology now allows high-quality genomes to be obtained for diverse taxa. However, minute metazoans pose a number of challenges to long-read sequencing. Standard protocols for long-read sequencing require a large input of high molecular weight (hmw) DNA—in the order of a microgram—which in turn requires larger amounts of fresh or well-preserved input tissue. Pooling individuals from field-collected specimens is often not possible and not desirable: many species cannot be captured in sufficiently large numbers, and pooling individuals complicates assembly by increasing genetic heterogeneity and bears the risk of mixing cryptic species. Small animals often need to be preserved as soon as they are removed from their natural habitats. Furthermore, to be precisely identified, individuals have to be sorted, prepared, and observed under a microscope. This results in delays between specimen collection and DNA extraction and cannot be done on living specimens. Therefore, most small metazoan species will have to be genome-sequenced from single, field-preserved specimens.

Recent progress has already decreased the amount of DNA needed for long-read sequencing. Kingan et al. genome-sequenced a single mosquito on the Pacific Biosciences (PacBio) platform [3]. Adams et al. obtained a chromosome-level assembly from a single, laboratory-bred, fruit fly based on a combination of Nanopore long reads, Illumina short reads, and low-input Hi-C sequencing [4]. However, most metazoans are even smaller than a single mosquito or fruit fly and would not yield the amount of DNA required by the applied protocols.

**Figure 1:**
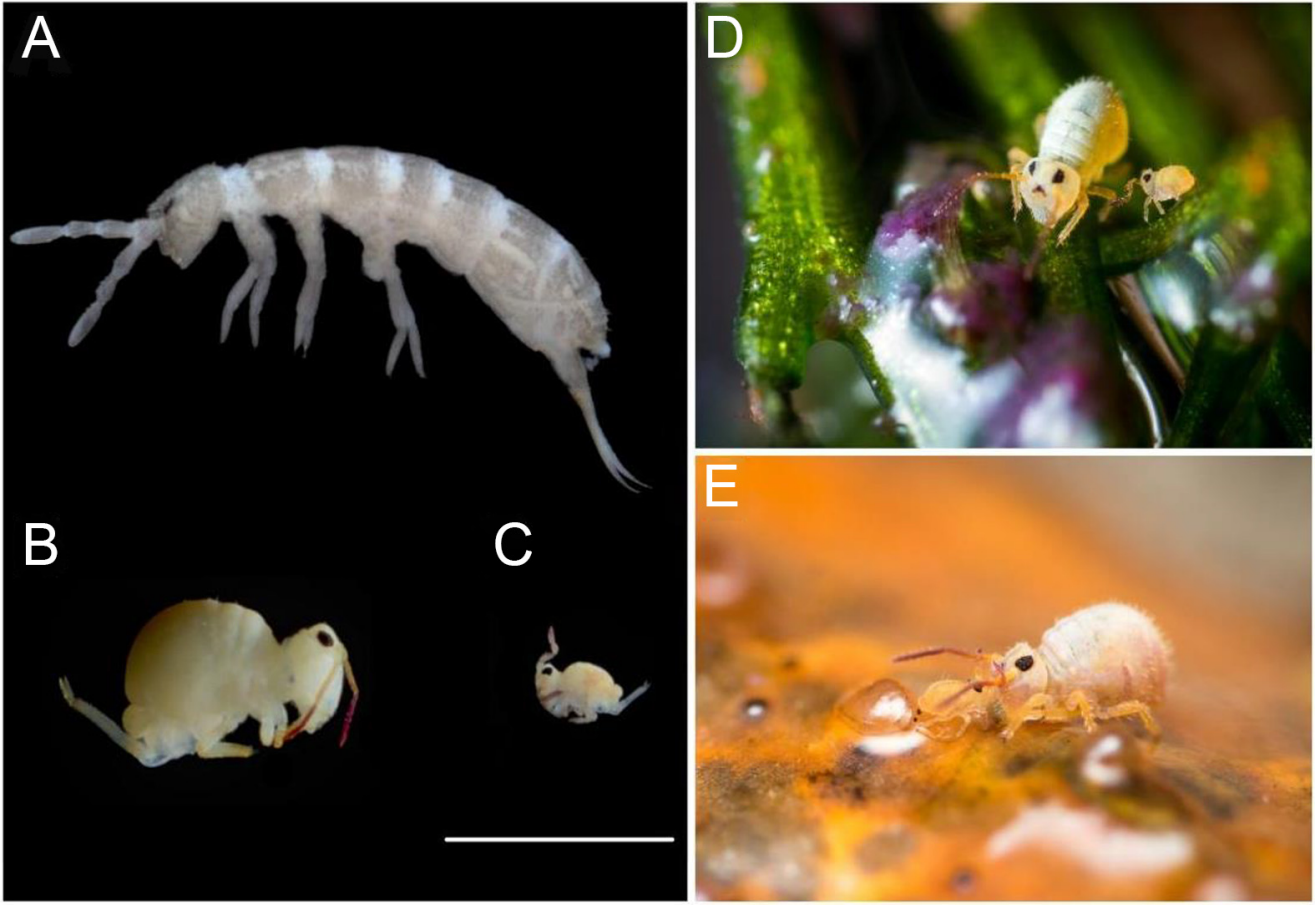
(A) *Desoria tigrina*. (B–E) *Sminthurides aquaticus* (B) female, (C) male, (D) male and female on wet plant, (E) courtship on a floating dead twig: the male uses its clasping antennae to grab the antennae of the much bigger female. (A–C) Specimens preserved in 96% ethanol, scale bar = 1 mm.

Here we present high-quality genomes of 2 non-model field-collected Collembola species (Arthropoda: Collembola): *Desoria tigrina* (length: 2 mm; Fig. 1A; NCBI:txid370036) and *Sminthurides aquaticus* (length: 1 mm; Fig. 1B–E; NCBI:txid281415). We extracted DNA from single specimens, preserved for 3–45 days in 96% ethanol, and used a recent whole-genome amplification-based ultra-low DNA input workflow for Single Molecule, Real-Time (SMRT) Sequencing (PacBio) [5] to produce libraries from as little as 5 ng DNA input. Using these libraries, we sequenced 1 SMRT cell for each species. To set the genomes as reliable references, we followed a thorough taxonomic workflow leading to the designation of a needed neotype for *S. aquaticus*. We investigated the resulting genomes for the presence of a β-lactam antibiotic synthesis pathway, an exceptional trait in the metazoan kingdom known in some species of edaphic Collembola [6]. We placed the 2 species in a genome-based phylogeny of Collembola. The resulting genomes are highly contiguous and nearly complete. The *S. aquaticus* assembly even has the highest contiguity compared to the Collembola genomes sequenced so far from hundreds of cultured specimens [7,8].

Thus, we show that high-quality, *de novo* genomes can be sequenced following a typical taxonomic workflow, even from submillimeter species that have been preserved for several days in 96% ethanol. This novel approach will add to the aim of biodiversity genomics to sequence all life on Earth, and make closer the day when whole-genome sequencing will be a routine component of integrative taxonomy.

## Materials and Methods

### Sequenced species

The collembolan *D. tigrina* (Entomobyomorpha, Isotomidae) is a hemiedaphic species: it is found in the upper layer of soil and litter. It is mostly found in anthropized environments [9]. It can be very abundant in vegetal compost, is found in crop fields [10], and can occur in caves as a troglophile [11]. In Western Europe it remains active in winter. The collembolan *S. aquaticus* (Symphypleona, Sminthurididae) is an epigeous, hygrophilous species that is widely spread in the Holarctic region [12]. Specimens often gather on plants, wood, and rocks emerging from the water surface. The animals can walk and jump on water surfaces thanks to elongated claws and a strong furca (jump appendage) with a tip that functions as a paddle on the surface tension. The species has a remarkably pronounced sexual dimorphism: the male is significantly smaller than the female and its modified antennae in the form of a prehensile organ allow it to clasp the female antennae in a courtship dance preceding external fecundation (Fig. 1B–E).

### Specimen collection and preparation

*Desoria tigrina* was collected in a garden compost bin 8 31.278 E, 50 8.358 N, 14 December 2019). Specimens were extracted from the compost with a Berlese funnel directly into 96% ethanol. DNA extraction was performed within ∼72 h. *Sminthurides aquaticus* was collected from a pond in a public garden 2 23.994 E, 48 51.534 N, 27 October 2019). Specimens were caught manually by eye using a small net and mouth-aspirator. They were preserved in 96% ethanol, kept at ambient temperature for 1 day until they could be stored at −20°C. They remained in cold storage for 1.5 months until we could proceed with DNA extraction. For each species, we gathered a pool of specimens collected simultaneously and pre-identified them all using a stereomicroscope (≤60× magnification). For *D. tigrina*, 4 specimens were used for DNA extraction (involving their destruction) and 30 were used for precise morphological identification. For *S. aquaticus*, 8 specimens were used for DNA extraction and 17 for morphological identification. Specimens used for morphological identification were cleared in lactic acid and potassium hydroxide, and they were mounted in permanent slides using Marc-André II mounting medium. Observations were made using a Leitz Wetzlar Diaplan with phase contrast, at 400–1,000× magnification.

### Ultra-low input PacBio sequencing

**Figure 2:**
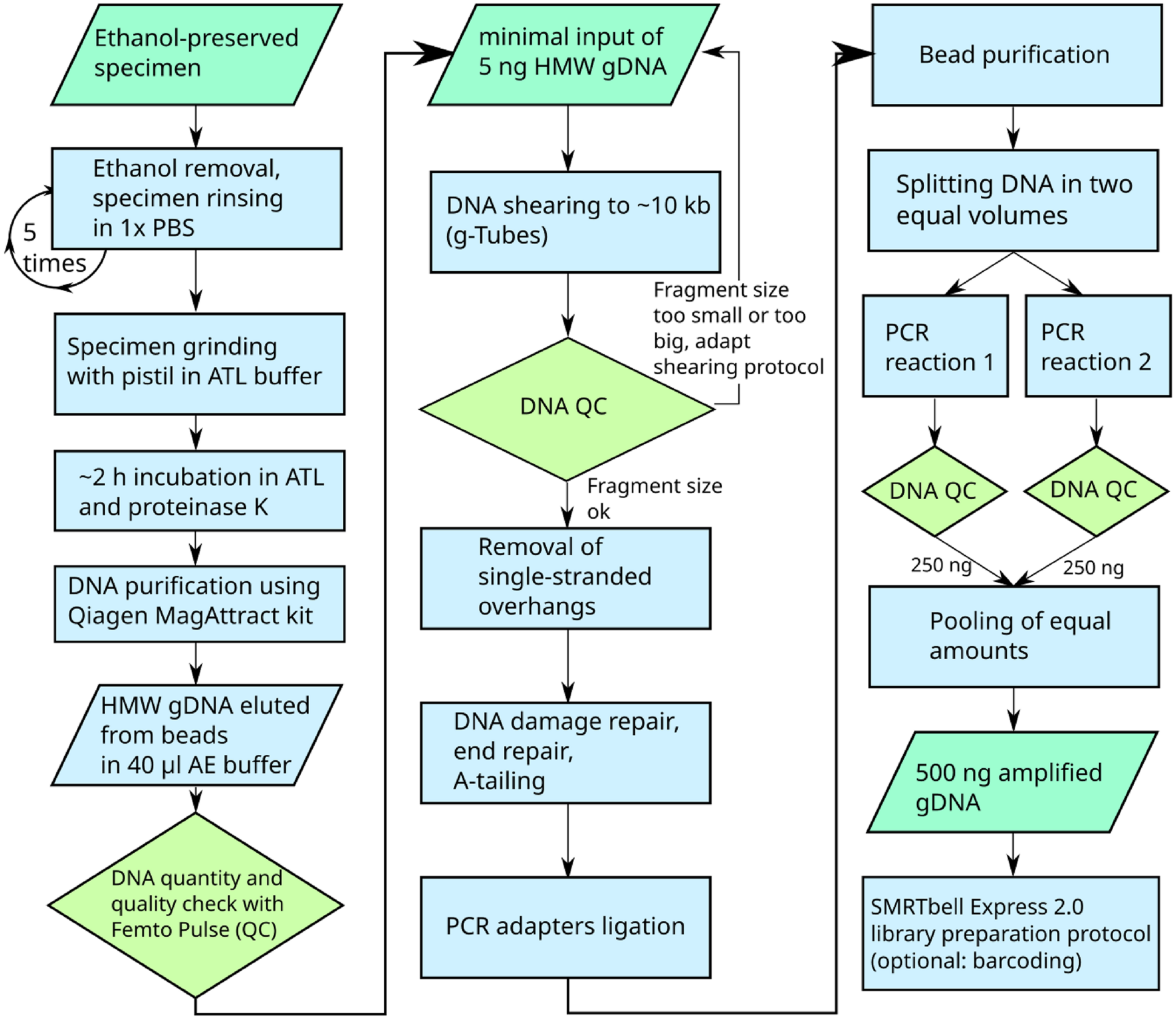
Flow chart of DNA extraction and ultra-low input workflow for SMRTbell Express 2.0 library preparation, for a single ethanol-preserved specimen. gDNA: genomic DNA; QC: quality control.

Our workflow for DNA extraction and ultra-low DNA input follows the flow chart shown in Fig. 2. Extraction was performed from a single specimen for both species. Specimens were rinsed in 1× phosphate-buffered saline (PBS) to remove residual ethanol. The solution was replaced 4 times with fresh PBS. Specimens were crushed by 1-way pistons, then DNA was extracted using the Qiagen MagAttract kit (Hilden, Germany). DNA was eluted once in 40 µL AE buffer. We performed 8 individual extractions from *S. aquaticus* and 4 individual extractions from *D. tigrina* specimens. Each DNA extract was quantified with the Quantus dsDNA system (Promega) and DNA quality was assessed with FEMTOpulse (Agilent). Randomly 1 DNA extract was selected for each species. The selected extract contained 59.24 ng hmw DNA (*D. tigrina*) and 16.16 ng hmw DNA (*S. aquaticus*), respectively (FEMTOpulse measurements are provided in Supplementary File S1).

Libraries were prepared using an early access kit for the Ultra-Low DNA Input Workflow for SMRT Sequencing (PacBio) [5]. Of the genomic hmw DNA extracts, 5 ng was fragmented with g-Tubes (Covaris) in an Eppendorf 5424 R centrifuge for 2 min at 1,902*g*. The resulting fragment sizes were again inspected with FEMTOpulse (Agilent). Next, single-stranded overhangs were enzymatically removed, followed by DNA damage repair, repair of DNA ends, and an A-tailing step. Double-stranded DNA adapter with a T-overhang was ligated for 1 h at 20°C and the resulting products were bead purified (ProNex, Promega, Madison, Wisconsin, USA), eluted, and split into 2 identical aliquots. DNA fragments with adapters were amplified in 2 different PCR reactions (Reaction 1: 98°C for 45 s, 14 cycles: 98°C for 10 s,  62°C for 15 s,  72°C for 7 min, final elongation 72°C 5 min; Reaction 2: 98°C for 30 s, 14 cycles: 98°C for 10 s,  60°C for 15 s,  68°C for 10 min, final elongation 68°C 5 min). PCR reactions were again bead purified and eluted in EB. Library fragments were assessed for quantity Quantus, Promega) and quality (FEMTOpulse, Santa Clara, California, USA). PCR fragments from both reactions were pooled in equal concentrations to achieve a total of 500 ng input for library preparation. Libraries were prepared following the low DNA input workflow for SMRT Sequencing (PacBio, Menlo Park, California, USA). Libraries were annealed to a sequencing primer (V4), bound to Sequel II DNA polymerase 2.0 with Binding kit 2.0, and sequenced in a Sequel II 8M SMRT cell for 30 h.

### Genome assembly

Generation of circular consensus sequencing (CCS) reads and adapter trimming was done in PacBio SMRTLink 8 with default parameters followed by deduplication of reads via pbmarkdup (v0.2.0 [13]) as recommended by PacBio. HiFi reads containing complete PCR adapter sequences were discarded. Genome properties were estimated with *k*-mer statistics prior to assembly. This is possible owing to the low error rates of HiFi reads. The *k*-mers were counted and aggregated using jellyfish 2.2.10 [14] (“jellyfish count -C -m 21 -t 20 -s 1000000000 -o jelly_k21.jf CCS.fasta” and “jellyfish histo -t 10 jelly_k21.jf > kmer.histo”) (Jellyfish, RRID:SCR_005491). We used GenomeScope 1.0 (GenomeScope, RRID:SCR_017014) [15] to estimate genome length, level of duplication, and heterozygosity through the web application [16].

Several long-read assembly tools were compared: FALCON (falcon-kit v1.8.0) (Falcon, RRID:SCR_016089) [17], Flye (v2.9.1-b1676) (Flye, RRID:SCR_017016) [18], HiCanu (v2.1) [19], Hifiasm (v0.12-r304) [20], IPA (v1.1.2) [21], and wtdbg2 v2.5 (WTDBG, RRID:SCR_017225) [22]. The command lines and statistics of preliminary assemblies are provided in Supplementary File S1. We retained Hifiasm, which produced the assemblies with significantly higher N50 for both species. We repeated the assembly process with Hifiasm after removing 5% and 10% of the shortest reads. Further separation of haplotigs was performed using alternatively purge_dups [23] (v1.0.1) or purge_haplotigs [24] (v1.1.0), and for each species we retained the method that achieved better BUSCO deduplication. The effect of purging on genome completeness was assessed with BUSCO v4.1.4 (BUSCO, RRID:SCR_015008) [25], in genome mode and with the “long” option, with the arthropoda_odb10 dataset [26]. For each species, we selected the method that led to the highest N50 and optimal purging (lowest amount of duplicated BUSCOs without significant loss of complete BUSCOs). For additional polishing of the resulting assemblies, we followed PacBio guidelines [17]. We used racon (Racon, RRID:SCR_017642) [27] (v1.4.10, parameter: “-u”) in combination with samtools (SAMTOOLS, RRID:SCR_002105) [28] (V1.9, parameters: “view -F 1796 -q 20”) and pbmm2 [29] (v1.1.0, parameters: “–preset CCS –sort”), a wrapper of minimap2 (Minimap2, RRID:SCR_018550) [30].

To assemble the mitochondrial genomes, we gathered CCS containing exclusively mitochondrial sequences, used blastn (blastn+ suite v2.10.0; RRID:SCR_001598) [31], and assembled them using Geneious 2020.1.2 (Geneious, RRID:SCR_010519) [32]. Circularity was validated manually, and nucleotide bases were called with a 75% threshold consensus. The mitochondrial genomes were annotated with the MITOS2 web server [33]. Coding DNA sequences were checked and corrected using Geneious to ensure that the presence of uncommon start codons and incomplete stop codons did not mislead the automatic annotation algorithms. Boundaries of the recombinant DNAs were slightly adjusted to make them contiguous with the *tRNA(val)*gene.

We used blastn to identify insertions of the mitochondrial genome in the nuclear genome (NUMTs). For this query, we used a 2× duplicated sequence of the mitochondrial genome to handle circularity. We recognized the presence of almost complete copies of the mitochondrial genome in the nuclear genomes of both species. We investigated the mapping of the CCS to the assembly in those locations using IGV [34] (v2.8.13) and recognized that in 1 instance, a misassembly occurred through the soldering of 2 NUMTs with CCS of mitochondrial origin. All CCS aligning with those 2 NUMTs were gathered with blastn and reassembled using Geneious. We could not find unambiguous NUMTs CCS (i.e., CCS carrying both nuclear and mitochondrial sequence) that would support the original assembly connection, and therefore we split the contig.

### Contamination control

We checked the assemblies for potential contamination from other organisms by querying the contigs against the NCBI database using protein-based (DIAMOND [35]) and nucleotide-based (blastn) alignments. Results were merged with Blobtools2 [36] (v2.3.3) using the “bestsum” algorithm. Contigs explicitly assigned to another lineage than metazoan were excluded from the assembly. Contigs assigned to Chordata were checked for presence of Arthropoda BUSCO. If Arthropoda BUSCOs were confirmed on such contigs, we retained them for the assembly.

### Assembly assessment

Curated assemblies were again evaluated with BUSCO (same parameters as before). We mapped the CCS on the assemblies using backmap [37] (v0.3), a perl wrapper of minimap2 and QualiMap2 [38]. Minimap2 was run with “-H -ax asm10” to map CCS on the assembly. We then performed another estimation of the genome size by dividing the number of mapped nucleotides by mode of the coverage distribution [37].

### Comparison with previous long-read assemblies

We compared our new genomes sequenced to previous Collembola assemblies that were generated with long-read and sometimes additional short-read data [7, 8, 45]. We also compared our Collembola assemblies to the draft genomes of 2 larger insects [3,39] (4 and 20 mm), which were also sequenced from single specimens but with the PacBio low-input workflow [40] (amplification-free).

### Alternative haplotig assembly

For both species, we obtained the alternative haplotig assembly by concatenating the alternate haplotig produced by Hifiasm with the duplicated contigs identified in the primary assembly during the purging step. We then further curated the alternative haplotig assembly by using sequentially Purge_dups and the decontamination strategy described above; and finally evaluated the BUSCOs completeness.

### Genome annotation

The primary assemblies were annotated with *ab initio* gene prediction. Repetitive regions were masked with RepeatModeler (RepeatModeler, RRID:SCR_015027) [41] (v2.0.1) with the options: “-LTRStruct -engine ncbi” using RepeatMasker (RepeatMasker, RRID:SCR_012954) [42] (open-4.0.9, options: “-xsmall -gff -nolow”). Protein sequences were predicted with AUGUSTUS (Augustus, RRID:SCR_008417) [43] (v3.3.3, option: “–softmasking = on”) reusing the BUSCO training results. Functional annotations were obtained by a local installation of eggNOG-mapper [44] (v2.0.1, option: “-m diamond”). If emapper recovered no annotations, we denoted sequences as “hypothetical protein” (for proteins without hits in emapper) or “uncharacterized protein” (for proteins with hits without annotations). To determine whether *D. tigrina* and *S. aquaticus* share the β-lactam synthesis gene found in some other Collembola, we searched the genomes for genes homologous of the isopenicillin N synthase (*IPNS*) and δ-(L-α-aminoadipoyl)-L-cysteine-D-valine synthetase (*ACVS*) genes of *Folsomia candida*. Those 2 genes belong to the same gene cluster. We used blastn: blastn and megablast to query the DNA sequences and tblastn to query the protein sequences against the combined primary and alternative haplotig assemblies. We also used blastp to query the protein sequences against the predicted protein sequences from the primary assemblies. The NBCI accession numbers of the searched sequences are *IPNS*—JX270832.1, *ACVS*—OXA60265.1.

### Phylogenetic analysis

We gathered 13 Collembola genome assemblies [7,8, 45, 46] from NCBI. For the outgroup, we selected a Diplura [47] and a Diptera [3] genome assemblies. The species list and the genome accession numbers are provided in Table 1. We used BUSCO v4.0.6 in short mode to search for orthologs, restricting the search to the arthropoda_odb10 dataset. We screened the obtained BUSCO sets to identify genes shared among the species, allowing only genes found for ≥75% of the species. We aligned single protein sequences with MAFFT (MAFFT, RRID:SCR_011811) [48] (v7.450), concatenated the alignments with FASconCAT-G [49] (v1.04), and trimmed the final alignment with trimAl (trimAl, RRID:SCR_017334) [50] (v1.2). We calculated a maximum likelihood tree with IQtree [51] (v1.6.12) with 1,000 non-parametric bootstrap replications.

**Table 1:** Species included in the phylogenetic analysis (taxonomic dataset expanded from [44])

Species	Order	Family	Repository	Accession	Source
*Anopheles coluzzii*	Diptera	Culicidae	NCBI	ASM413651v2	[3]
*Catajapyx aquilonaris*	Dicellurata	Japygidae	NCBI	GCA_000934665.2	[47]
*Ceratophysella communis*	Poduromorpha	Hypogastruridae	NCBI	GCA_009869905.1	[44]
*Desoria tigrina*	Entomobryomorpha	Isotomidae	EMBL-ENA	ERZ1473261	This study
*Folsomia candida*	Entomobryomorpha	Isotomidae	NCBI	GCA_002217175.1	[7]
*Lipothrix lubbocki*	Symphypleona	Sminthuridae	NCBI	GCA_009872335.1	[44]
*Mesaphorura yosii*	Poduromorpha	Tullbergiidae	NCBI	GCA_009869945.1	[44]
*Neelides* sp.	Neelipleona	Neelidae	NCBI	GCA_009869795.1	[44]
*Oncopodura yosiiana*	Entomobryomorpha	Oncopoduridae	NCBI	GCA_009869805.1	[44]
*Orchesella cincta*	Entomobryomorpha	Entomobryidae	NCBI	GCA_001718145.1	[45]
*Pseudachorutes palmiensis*	Poduromorpha	Neanuridae	NCBI	GCA_009869845.1	[44]
*Pseudobourletiella spinata*	Symphypleona	Bourletiellidae	NCBI	GCA_009870155.1	[44]
*Pygmarrhopalites habei*	Symphypleona	Arrhopalitidae	NCBI	GCA_009870185.1	[44]
*Sinella curviseta*	Entomobryomorpha	Entomobryidae	NCBI	GCA_004115045.1	[8]
*Sminthurides aquaticus*	Symphypleona	Sminthurididae	EMBL-ENA	ERZ1473260	This study
*Sminthurides bifidus*	Symphypleona	Sminthurididae	NCBI	GCA_009872375.1	[44]
*Thalassaphorura encarpata*	Poduromorpha	Onychiuridae	NCBI	GCA_009869925.1	[44]
*Tomocerus qinae*	Entomobryomorpha	Tomoceridae	NCBI	GCA_009869885.1	[44]

## Results

### Species biology and taxonomy

In terms of biomass and number *D. tigrina* was by far the dominant Collembola found in the compost bin during the winter season. Morphological observations placed the collected specimens unambiguously within the *D. tigrina* group [52]. Within this group, outer maxillary palp chaetotaxy was used to distinguish *D. tigrina* from its sibling species *Desoria grisea* following Fjellberg [52]. Identification was further validated following Potapow [9]. Six females, 2 males, and 2 juveniles on 4 slides numbered EA013940–3 were deposited in the Apterygota collection of the National Museum of Natural History, Paris. Seventeen females and 3 males on 12 slides labelled CSCH-1326–37 were deposited in the Apterygota collection at Senckenberg, Görlitz.

*Sminthurides aquaticus* was the only Collembola forming a population on the pond at the site of collection (i.e., no accidental fall on water surface). Abundantly found in October 2019, it was observed again in June 2020 in large numbers and with courtship behavior undergoing (Fig. 1D and E). The specimen identification was unambiguous following [12,52,53]. One male on a slide numbered EA060001 is designated as the neotype for *S. aquaticus* (see discussion) and was deposited in the Apterygota collection at the National Museum of Natural History, Paris, along with 3 females and 2 males on 5 slides (EA060002–6) and 20 individuals in 96% ethanol (CS.371, leg. C. Schneider). Two males, 3 females, and 1 juvenile on 5 slides numbered CSCH-1344–8 were deposited in the Apterygota collection at Senckenberg, Görlitz.

### DNA sequencing

**Figure 3:**
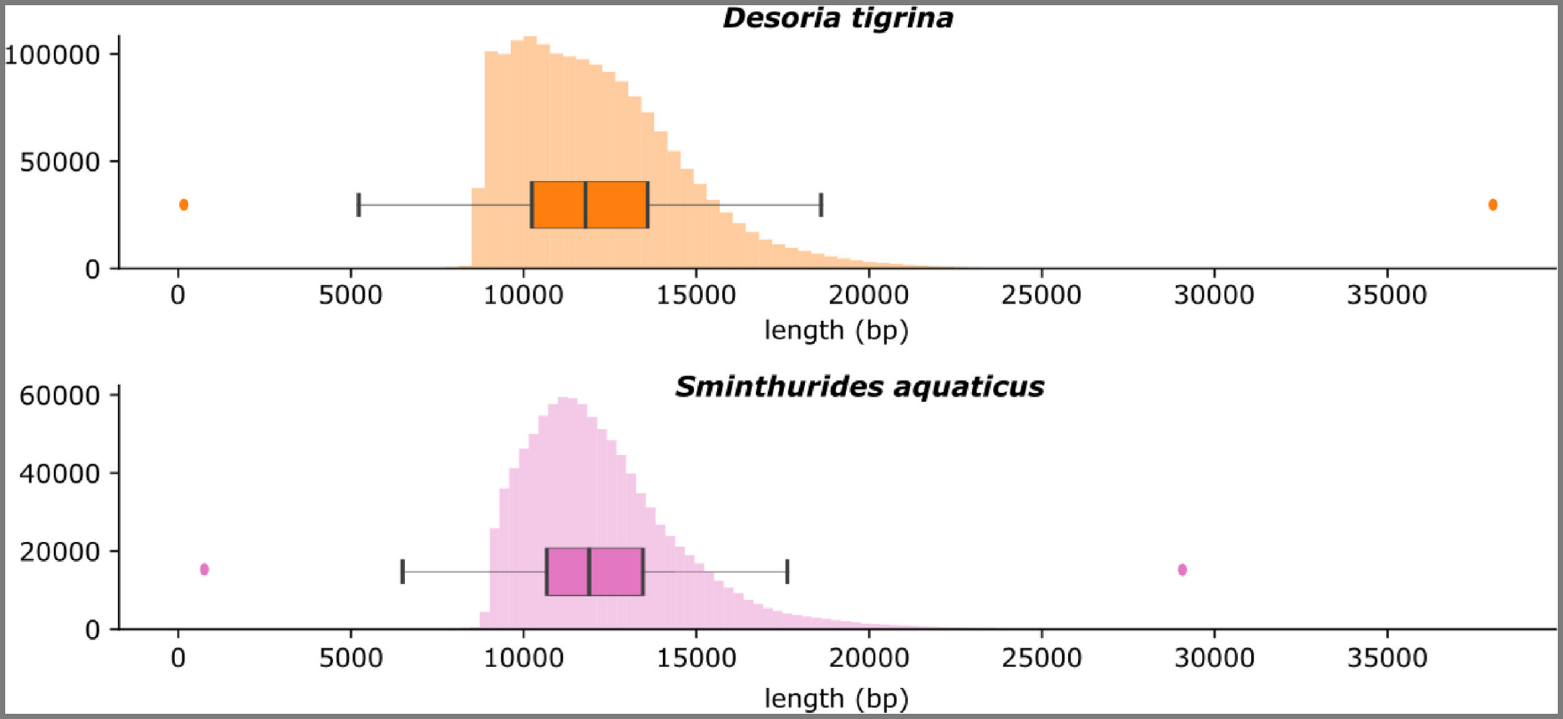
Distribution of CCS length. Outliers are not shown on the boxplot, except minimum and maximum length values each represented by a dot.

For *D. tigrina* a total of 20.22 Gb HiFi data (Q ≥ 20) were generated, with mean read length of 12,155 bp, median read length of 11,792 bp, and maximum read length of 37,982 bp. The distribution of read length is reported in Fig. 3. From the *k*-mer content of the reads, the genome haploid length was estimated to be ∼168 Mb with 1.43% of heterozygosity and 3% duplications.

For *S. aquaticus* a total of 12.4 Gb HiFi data (Q ≥ 20) were generated with mean length of 12,308 bp, median read length of 11,893 bp, and maximum read length of 29,073 bp. The distribution of read length is reported in Fig. 3. From the *k*-mer content of the reads, the genome haploid length was estimated to be ∼152 Mb with 0.96% of heterozygosity and 0.78% duplications.

### Genome assembly

Overall, Hifiasm produced the best assemblies for both species (Supplementary File S1).

**Figure 4:**
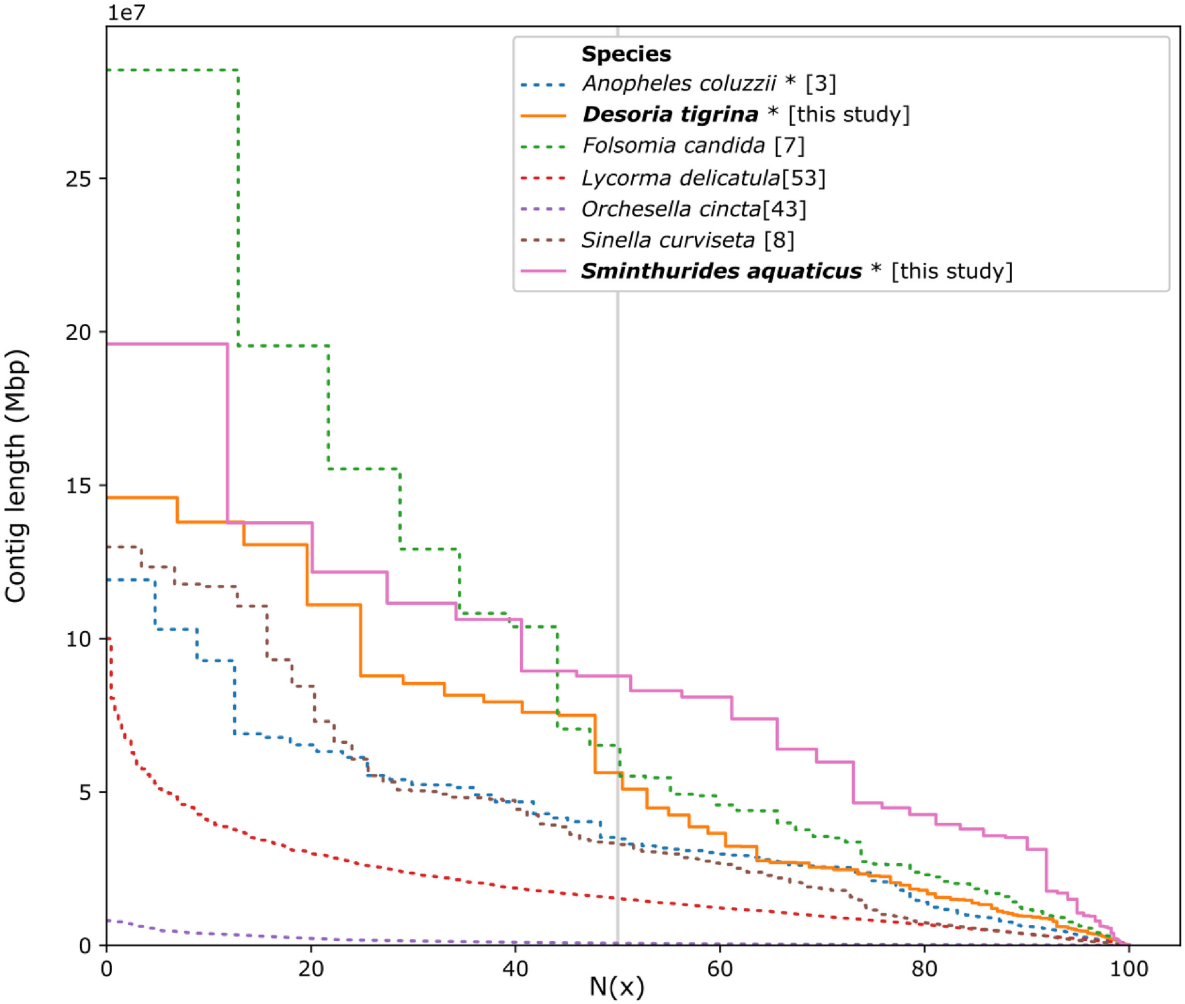
N(x) plot of recent high-quality genomes assembled with long reads, including the assemblies presented in this study.

For *D. tigrina*, the most contiguous assembly (Table 2, Fig. 4) was obtained by selecting 95% of the reads excluding the shortest one. Purging haplotigs with Purge_dups resulted in fewer duplicated BUSCOs than with Purge_haplotigs. No contigs were found to be of non-metazoan origin. While some contigs were assigned to Chordata taxa, they all carried Arthropoda-specific BUSCOs and were therefore kept. The curated primary assembly of *D. tigrina* is composed of 142 contigs and has a size of 211,462,971 bp and an N50 value of 5.63 Mb (Table 2, Fig. 4). Mean coverage is 95.40×, with a coverage distribution mode of 103×. The genome size is 196 Mb, estimated from mapped reads and coverage. BUSCO search on the whole assembly yielded 96% complete (C) BUSCOs (including 1.7% duplicated [D]), 0.9% fragmented (F) BUSCOs, and 3.1% missing (M) BUSCOs. The mitochondrial genome assembly was complete, for a size of 15,139 bp. Two large NUMTs were found, each on a different contig. One was 18,113 bp (120% of the mitochondrial genome size) and the other was 28,173 bp (186% of the mitochondrial genome size). Examination of the mapped reads revealed no obvious misassembly for the smaller NUMT (spanned by reads that contained mitochondrial and genomic sequence), but the larger NUMT was bridged in the middle by reads containing exclusively mitochondrial sequence. Therefore, we split the contig carrying the larger NUMT, keeping on each side a partial NUMT sequence supported by reads containing mitochondrial and genomic sequence. The alternative haplotig assembly of *D. tigrina* is composed of 1,611 contigs and has a size of 189,752,789 bp and an N50 value of 0.27 Mb; BUSCO search on the alternative haplotig assembly yielded 89% complete (including 3.8% duplicated), 0.9% fragmented, and 10.1% missing BUSCOs.

**Table 2:** Statistics of several assemblies generated from long-read sequencing (with or without additional short reads) and/or low-input approach

Parameter	*Desoria tigrina*	*Sminthurides aquaticus*	*Folsomia candida*	*Orchesella cincta*	*Sinella curviseta*	*Anopheles coluzzii*	*Lycorma delicatula*
Class	Collembola	Collembola	Collembola	Collembola	Collembola	Insecta	Insecta
Body size class	2 mm	1 mm	2 mm	2 mm	2 mm	4 mm	20 mm
No. specimens in input	1 (PacBio)	1 (PacBio)	1,600 (Pacbio) + 100 (Illumina)	40 (PacBio) + 1 (Illumina)	500 (PacBio) + 10 (Illumina)	1 (PacBio)	1 (PacBio)
WGA	Yes	Yes	No	No	No	No	No
No. contigs	142	79	162	9,398	599	1,034	2,927
Largest contig (bp)	14,592,742	19,603,089	28,534,321	807,113	12,986,801	11,911,669	9,998,986
Total length (bp)	211,462,971	165,915,169	221,702,752	286,764,906	381,458,724	340,555,854	2,252,044,789
N50 (bp)	5,628,779	8,776,828	6,519,406	65,879	3,284,409	2,625,112	1,519,606
N75 (bp)	2,264,114	4,641,270	2,726,164	23,461	1,147,450	440,054	811,306
L50 (bp)	11	7	8	925	32	36	434
L75 (bp)	28	13	21	2,812	74	126	935
BUSCO % C (D), F, M	96 (1.7), 0.9, 3.1	96.1 (1.6), 1.3, 2.6	97.1 (0.9), 0.5, 2.4	94.5 (3.2), 1.8, 3.7	95.6 (4.4), 1.3, 3.1	99.6 (2.9), 0.0, 0.4	96.5 (1.9), 2.0, 1.5
Source	This study	This study	[45]	[7]	[8]	[3]	[39]
Assembly accession	EMBL-ENA: PRJEB39696	EMBL-ENA: PRJEB39696	NCBI: ASM221717v1	NCBI:ASM171814v	NCBI:ASM411504v1	NCBI: ASM413651v2	doi:10.15482/USDA.ADC/1503745

For *S. aquaticus* the best assembly was obtained by using all the reads (Table 2, Fig. 4). Purging haplotigs with Purge_haplotigs resulted in fewer duplicated BUSCOs than with Purge_dups. Two contigs (totaling 243,436 bp) were found to be from a fungus and a cyanobacterium, respectively, and were removed. Some contigs were assigned to Chordata taxa but all of those carried Arthropoda-specific BUSCOs and were kept. The curated primary assembly of *S. aquaticus* is composed of 79 contigs and has a size of 165,915,169 bp and an N50 value of 8.78 Mb (Table 2, Fig. 4). Mean coverage is 72.67×, and coverage distribution mode is 77×. The genome size is 157 Mb, estimated from mapped reads and coverage. BUSCO search on the whole assembly yielded 96.1% complete BUSCOs (including 1.6% duplicated), 1.3% fragmented BUSCOs, and 2.6% missing BUSCOs. The mitochondrial genome assembly was complete, for a size of 16,099 bp. A large NUMT was detected in 1 of the purged contigs (haplotigs), but none were found in the primary contigs, so we decided not to investigate further. Several small contigs were found to be assembled from mitochondrial reads and were removed. The alternative haplotig assembly of *S. aquaticus* is composed of 459 contigs and has a size of 150,171,336 bp and an N50 value of 1.00 Mb; BUSCO search on the alternative haplotig assembly yielded 87.5% complete (including 2.9% duplicated), 1.4% fragmented, and 2.6% missing BUSCOS.

### Comparison with previous long-read assemblies

In terms of BUSCO completeness scores, our assemblies are comparable to previous high-quality Collembola genomes assembled from a large pool of specimens (95.8% and 96.1% vs 94.5⁠–97.1% complete; Table 2). In terms of assembly contiguity, our *S. aquaticus* has the highest and the *D. tigrina* assembly has the third-highest contig N50 value (Table 2, Fig. 4). The insect genomes obtained sequencing from single specimen using the PacBio low-input workflow have higher BUSCO scores (96.5% and 99.6%) but lower contiguity (Table 2, Fig. 4). Together, this shows that assemblies generated with the ultra-low input workflow and long-read sequencing can reach or surpass the level of quality of assemblies obtained with the standard or low-input workflow.

### Genome annotation

In the mitochondrial genome of both species, we identified the complete set of 37 mitochondrial genes (13 proteins, 22 transfer RNA, and 2 ribosomal RNA coding genes) typically found in Hexapoda. In the nuclear genome, we predicted 24,423 proteins for *D. tigrina*, 15,546 (63.65%) of which had homologs in other organisms and 8,877 were labeled as “hypothetical protein.” BUSCO search on the predicted proteins yielded 96.2% complete BUSCO including 2.6% duplicated, 1.2% fragmented, and 2.6% missing. For *S. aquaticus*, we predicted 17,624 proteins in the nuclear genome, 11,989 (68.03%) of which had homologs in other organisms and 5,635 were labeled “hypothetical protein.” BUSCO search on the predicted proteins yielded 95.3% complete BUSCO including 2.1% duplicated, 1.6% fragmented, and 3.1% missing.

### β-lactam biosynthetic pathway

Collembola exhibit a diversity in the presence of a β-lactam antibiotic synthesis pathway, which is secondarily lost in some species. Therefore, we analyzed our genomes for the presence of a β-lactam antibiotic synthesis pathway. No homologs of the genes *IPNS* and *ACVS* could be identified in the 2 genomes, indicating the absence of the β-lactam antibiotic synthesis pathway in *D. tigrina* and *S. aquaticus*. However, by screening the functional annotation of the predicted genes, we identified 4 genes related to aminopenicillanic-acid-acyltransferase (penDE) in the genome of *S. aquaticus* and 5 penDE-like genes in the genome of *D. tigrina*.

### Phylogeny

**Figure 5:**
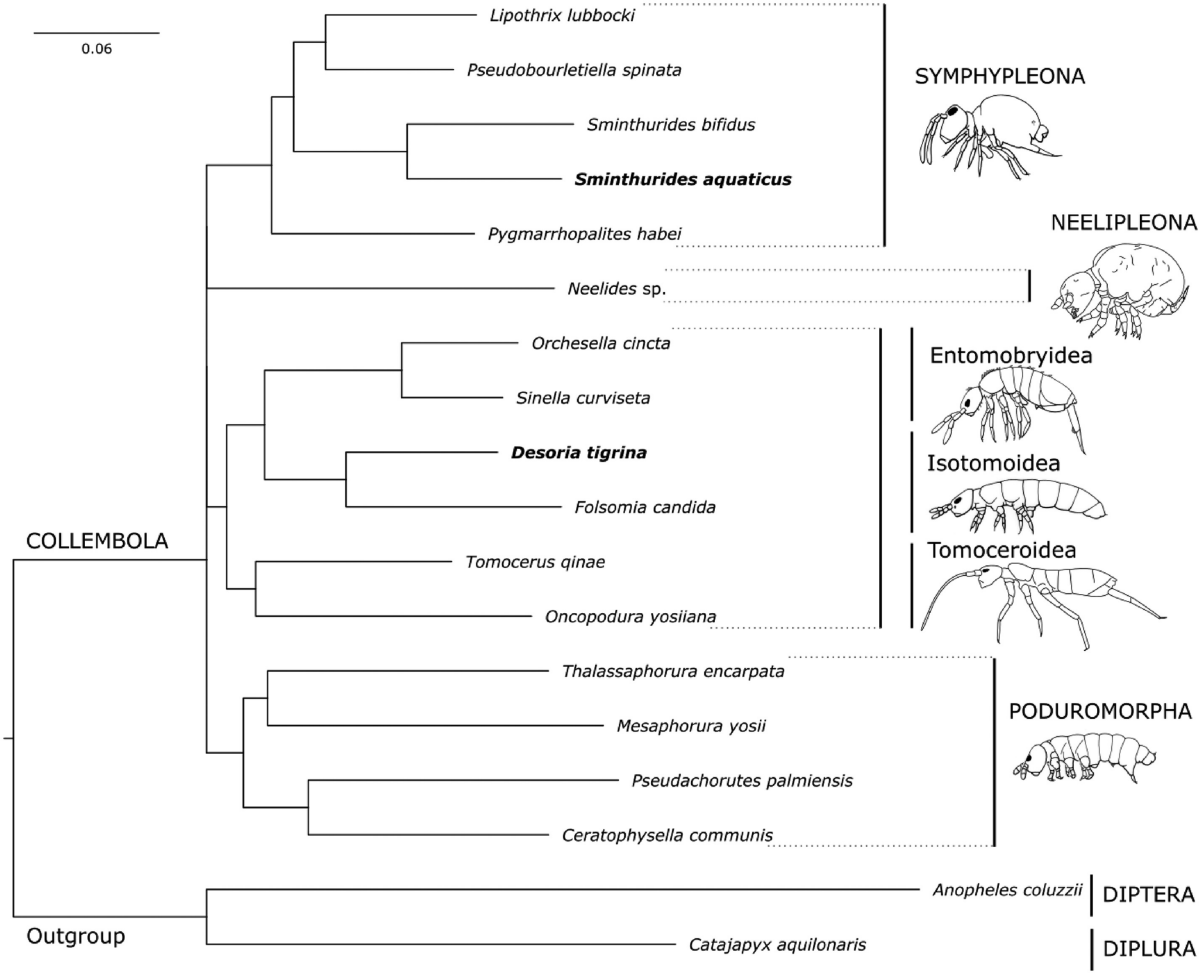
Phylogeny of Collembola based on the alignment of 545 protein sequences. Bootstrap support of shown nodes is 100%; nodes with bootstrap supports of 73% were collapsed.

To place our 2 species in a molecular phylogeny of Collembola, we used BUSCO genes as conserved phylogenetic markers. Allowing for a maximum of 25% missing sequence for each ortholog, we retained 545 complete BUSCOs to align. The total length of the trimmed alignments is 171,703 sites. We used IQTree to infer a phylogenetic tree, shown in Fig. 5. Our 2 newly sequenced species find their expected placement on the Collembola phylogeny with *Sminthurides aquaticus* as a sister species to *S. bifidus* (both are representives of the genus *Sminthurides*, family Sminthurididae) and *D. tigrina* as a sister species to *F. candida* (both are representatives of the family Isotomidae). Our tree also recovered the monophyly of orders Symphypleona, Poduromorpha, and Entomobryomorpoha with 100% bootstrap support. However, the basal relationships between the 4 orders of Collembola receive negligible bootstrap support (=73%), indicating phylogenetic irresolution. The rest of the tree is consistent with the to-date most detailed genome-based phylogeny of Sun et al. [46]. The >400 million years old basal relationships of Collembola have long been debated. They are sensitive to data sampling, and phylogenetic artifacts such as long branch attraction and random root occur [54]. Additional genomes of key Collembola representatives and more informative phylogenetic markers [46] are needed to properly address the problem of basal relationships within Collembola.

## Discussion

### Value of the ultra-low input workflow

Long-read sequencing as the future for *de novo* genome assembly has normally required larger amounts of input tissue, which limits its application to larger organisms. However, a substantial portion of biodiversity is represented by tiny species. Here, we address this important challenge in biodiversity genomics and provide a proof of concept that it is now possible to sequence high-quality reference genomes from field-collected individual tiny Collembola species. The 5 ng input of the PacBio ultra-low input workflow is a significant decrease from the 150 ng input required by the PacBio low-input workflow (whole-genome amplification [WGA]-free). And yet the ultra-low input still allows high-quality genomic data to be captured: our final assemblies were of high contiguity and completeness on par with recent genomes from larger insects sequenced using the low-input protocol [3,39]. Our new genomes are also on par with the previously best reference genomes for Collembola: *F. candida* and *S. curviseta*, which were DNA sequenced from hundreds of specimens maintained in culture [7,8]. *Sminthurides aquaticus* even achieved the highest N50 and N75 among the compared assemblies. The quality of the new assemblies makes us consider that there are even additional benefits in the ultra-low input protocol besides sequencing organisms too small for WGA-free approaches. For species that are not too small, it can be used to generate long-read data from a fraction of the total DNA. This could be levered to implement approaches combining long-read and Hi-C for even smaller species than a fruit fly [4]. This can also allow the sequenced specimen to be retained to serve as a voucher, by removing the need to crush the specimen to maximize hmw DNA recovery.

### Ensuring taxonomic quality

It is essential that a reliable reference genome be supported by a solid and revisable taxonomy, to be useful for any meaningful downstream analysis. Taxonomy quality has always been an issue of sequence databases [55, 56]. This is especially true for field-collected specimens from taxonomically poorly known groups that are often riddled with cryptic diversity and difficulty of species identification based on a few subtle characters. Therefore, we documented species collection and identification by morphological characters, provided macro photographs, and preserved co-captured specimens of the same species in the collections of 2 European museums. This way, we ensure the taxonomic traceability of the reference genome, which should be a prerequisite for any meaningful biodiversity genomics where species identification is not straightforward.

The genus *Desoria* has a complex taxonomy. Within the *D. tigrina* group sensu Fjellberg 2007 [52], *D. tigrina* and *D. grisea* are 2 sibling species, described in the early times of modern Collembola systematics. *Desoria grisea* was redescribed by Fjellberg [52] from its type locality. Fjellberg reported that the 2 species, while extremely similar, could be consistently distinguished by the organization of the labial palp chaetae. We examined 30 specimens from our collection spot and each of them were identified as *D. tigrina*, supporting the identity of the specimen used for sequencing.

*Sminthurides aquaticus* was originally described from France and has been recognized to be widely spread throughout the Holarctic region. We confirmed that all our collected specimens are identical to the accepted descriptions of *S. aquaticus*. The species was originally described by Bourlet in 1841 probably from the north of France. However, Bourlet did not make any reference to a type series and, to our knowledge, did not preserve any specimens. We consider that the population we sampled in Paris is suitable to provide a neotype for this species: the population is abundant, settled, and easily accessible for further studies. This also offers the uncommon opportunity to have a neotype closely related to the reference genome for the species.

### Heterozygosity

The higher level of heterozygosity in *D. tigrina* compared to *S. aquaticus* seems consistent with the expected level of isolation of the populations. *Desoria tigrina* invaded the compost that was set up 1 year before the collection. The species is very mobile, being rather large and equipped with a long furca, and gene flow must be active across the nearby surrounding fields and gardens. On the other hand, the sampled population of *S. aquaticus* seems rather isolated in a small area (artificial pond in a public garden).

### β-lactam synthesis in Collembola

Recent results from transcriptomes show that several edaphic species from the orders Poduromorpha and Entomobryomorpha can synthesize β-lactam antibiotics [6]. Two essential genes of the β-lactam synthesis pathway, *ACVS* and *IPNS*, are consistently found in 4 euedaphic species (“true” soil dweller) but missing in 2 of 4 hemiedaphic species (living in upper layer of soil, litter, and dead wood) and always missing in 7 atmobiotic species (species living on vegetation, freshwater surface, or tidal zone). The genes are absent from soil dwellers from the class Diplura and Protura, 2 close relatives of Collembola. The antibiotic biosynthesis likely resulted from a single horizontal gene transfer event with subsequent loss of antibiotic synthesis ability in some of the investigated species [6].

We report the absence of *ACVS* and *IPNS* in the genomes of *S. aquaticus* and *D. tigrina. Sminthurides aquaticus* belongs to a family of Symphypleona that was not investigated by Suring et al. [6]. So far, no Symphypleona are known to carry those genes, but it must be noted that none of the tested species are soil-dwelling species. The Symphypleona species in the Suring et al. [6] dataset are vegetation dwellers. Because *S. aquaticus* dwells on freshwater surfaces, our results support the lack of antibiotic production in semi-aquatic species. The absence of the genes in *D. tigrina* is rather unexpected because the species lives in organic-rich litter with potentially high microbial contents. After *F. candida, D. tigrina* is the second member of the large Isotomidae family to be investigated for antibiotic production. *Desoria tigrina* is in the same class size as *F. candida* but is expected to be more mobile owing to its more developed legs, furca, and eye-patch (*F. candida* is eyeless). This suggests that antibiotic synthesis is specific to a true soil-dwelling (euedaphic) lifestyle, and it might be lost by more mobile species.

### Antibiotic synthesis in Collembola

Both *D. tigrina* and *S. aquaticus* possess *penDE*-like genes. Such genes were also reported in *F. candida* [6]. The penDE is the last enzyme in the penicillin biosynthetic pathway of the fungi *Emericella nidulans*, and converts isopenicillin N (product of *INPS* activity) to penicillin G. In *F. candida*, the *penDE*-like gene does not belong to the β-lactam synthesis gene cluster. Homologs of *penDE* are also known in fungi that do not produce antibiotics. Suring et al. [6] suggest that penDE-like genes may have been co-opted for the completion of the penicillin synthesis in *F. candida* after the acquisition of the β-lactam synthesis gene cluster. Consequently, the presence of *penDE* in *S. aquaticus* and *D. tigrina* is not a solid indicator of a lost antibiotic production trait in these species. Altogether, the assumption that the horizontal gene transfer is an ancestral acquisition to Collembola should be taken with caution bucause the basal relationships between Collembola orders are still unresolved. For further elucidation, edaphic species of orders Symphypleona and Neelipleona should be investigated for the antibiotic production trait.

## Conclusions

The LOEWE-TBG excellence cluster supports the goal of the EBP, which aims to sequence all eukaryotic species. Although the first high-quality genomes were generated for species with easy access to abundant and fresh samples, similar high-quality genomes can now be generated for tiny taxa or taxa that are otherwise difficult to sequence. Most known eukaryotic biodiversity belongs to very small metazoans that in addition need to be preserved for some time before genome sequencing. Access to their genomes provides insights into the formation, maintenance, and functioning of eukaryotic biodiversity and presents new opportunities for natural resource management and bioprospecting. The ability to genome-sequence these species is essential for the success of biodiversity genomics initiatives. Our genomes sequenced from 5 ng DNA actually exceed the 1Mb N50 contig continuity required by the EBP project when >100 ng DNA are available. We are convinced that integrating high-quality genomics with the typical workflow of small, field-collected metazoans is an essential approach toward the creation of a solid reference genome database for millions of minute non-model species belonging to taxonomically challenging groups.

## Data Availability

The data underlying this article are available in the EMBL-ENA database and can be accessed with accession No. PRJEB39696 including: *S. aquaticus* CCS, curated primary assembly, and annotation with accessions Nos. ERR4407379, GCA_906901655; and *D. tigrina* CCS, curated primary assembly, and annotation with accession Nos. ERR4407422, GCA_906901685.

Supporting data, including primary and alternative haplotig assemblies, and annotation files, are deposited in the *GigaScience* database, GigaDB, for both *Sminthurides aquaticus* [57] and *Desoria tigrine* [58].

## Additional Files

**Supplementary File S1**: Report on preliminary assemblies, including assembly statistics and details of assembly tools and command lines.

## Abbreviations

BLAST: Basic Local Alignment Search Tool; bp: base pairs; BUSCO: Benchmarking Universal Single-Copy Orthologs; CCS: circular consensus sequencing; EBP: Earth BioGenome Project; Gb: gigabase pairs; hmw: high molecular weight; IGV: Integrative Genomics Viewer; kb: kilobase pairs; LOEWE-TBG: LOEWE Center for Translational Biodiversity Genomics; MAFFT: Multiple Alignment using Fast Fourier Transform; Mb: megabase pairs; NCBI: National Center for Biotechnology Information; NUMT: nuclear mitochondrial DNA; PacBio: Pacific Biosciences; SMRT: single molecule, real-time; WGA: whole-genome amplification.

## Competing Interests

The authors declare that they have no competing interests.

## Authors’ Contributions

C.S. conceived the project; C.S. and C.A.D'H. collected, identified, and photographed the specimens; B.H. performed the DNA extraction, the library preparation, and the sequencing; C.W. and C.S. assembled and analyzed the genomes; M.W. and A.J. contributed the phylogenomic analysis; C.S. and M.B. led the writing of the manuscript; C.G. performed experiments (not presented here) that helped steer the project and further advised on the study; and M.H. revised the manuscript. All authors read and approved the final manuscript for submission.

## Supplementary Material

giab035_GIGA-D-20-00364_Original_Submission

giab035_GIGA-D-20-00364_Revision_1

giab035_Response_to_Reviewer_Comments_Original_Submission

giab035_Reviewer_1_Report_Original_Submission Arong Luo -- 1/9/2021 Reviewed

giab035_Reviewer_2_Report_Original_Submission Mahul Chakraborty -- 1/19/2021 Reviewed

giab035_Supplemental_Files
