# Supplementary material for: Two high-quality de novo genomes from single ethanol-preserved specimens of tiny metazoans (Collembola)
Source: Gigascience. 2021 May 21;10(5):giab035. doi: 10.1093/gigascience/giab035 (PMC8138834; doi:10.1093/gigascience/giab035)
Supplement: giab035_GIGA-D-20-00364_Original_Submission [file giab035_giga-d-20-00364_original_submission.pdf]

GigaScience

Two high-quality de novo genomes from single ethanol-preserved specimens of tiny metazoans (Collembola).

--Manuscript Draft--

**Manuscript Number:**	GIGA-D-20-00364
**Full Title:**	Two high-quality de novo genomes from single ethanol-preserved specimens of tiny metazoans (Collembola).
**Article Type:**	Data Note
**Funding Information:**	
**Abstract:**	Background<br><br>Genome sequencing of all known eukaryotes on Earth promises unprecedented advances in biological sciences and in biodiversity-related applied fields such as environmental management and natural product research. Advances in long read DNA sequencing make it feasible to generate high-quality genomes for many non-genetic model species. However, long read sequencing today relies on sizable quantities of high-quality, high molecular weight (hmw) DNA which is mostly obtained from fresh tissues. This is a challenge for biodiversity genomics of most metazoan species, which are tiny and need to be preserved immediately after collection. Here we present de novo genomes of two species of submillimeter Collembola. For each, we prepared the sequencing library from hmwDNA extracted from a single specimen and using a novel Ultra-Low input protocol from Pacific Bioscience.<br><br>Results<br><br>The two assembled genomes have N50 values over 5.5 and 8.5 Mb respectively, and both contain ~96% of BUSCO genes. Thus, they are highly contiguous and complete. The genomes are supported by an integrative taxonomy approach including placement in a genome-based phylogeny of Collembola and designation of a neotype for one of the species. Higher heterozygosity values are recorded in the more mobile species. Both species are devoid of the biosynthetic pathway for beta-lactam antibiotics known in several Collembola, confirming the tight correlation of antibiotics synthesis with the species way of life.<br><br>Conclusions<br><br>It is now possible to generate high-quality genomes from single-specimens of minute, field-preserved metazoans, exceeding the minimum contig N50 (1Mb) required by the Earth BioGenome Project.
**Corresponding Author:**	Clément Schneider, Ph.D.<br>Senckenberg Gesellschaft fur Naturforschung<br>Görlitz, GERMANY
**Corresponding Author Secondary Information:**	
**Corresponding Author's Institution:**	Senckenberg Gesellschaft fur Naturforschung
**Corresponding Author's Secondary Institution:**	
**First Author:**	Clément Schneider, Ph.D.
**First Author Secondary Information:**	
**Order of Authors:**	Clément Schneider, Ph.D.
	Christian Woehle
	Carola Greve
	Cyrille A. D’Haese

Powered by Editorial Manager® and ProduXion Manager® from Aries Systems Corporation

	Magnus Wolf
	Michael Hiller
	Axel Janke
	Miklós Bálint
	Bruno Hüttel
**Order of Authors Secondary Information:**	
**Additional Information:**	
**Question**	**Response**
Are you submitting this manuscript to a special series or article collection?	No
**Experimental design and statistics**<br><br>Full details of the experimental design and statistical methods used should be given in the Methods section, as detailed in our Minimum Standards Reporting Checklist. Information essential to interpreting the data presented should be made available in the figure legends.<br><br>Have you included all the information requested in your manuscript?	Yes
**Resources**<br><br>A description of all resources used, including antibodies, cell lines, animals and software tools, with enough information to allow them to be uniquely identified, should be included in the Methods section. Authors are strongly encouraged to cite Research Resource Identifiers (RRIDs) for antibodies, model organisms and tools, where possible.<br><br>Have you included the information requested as detailed in our Minimum Standards Reporting Checklist?	Yes
**Availability of data and materials**<br><br>All datasets and code on which the conclusions of the paper rely must be	Yes

either included in your submission or deposited in publicly available repositories (where available and ethically appropriate), referencing such data using a unique identifier in the references and in the “Availability of Data and Materials” section of your manuscript.<br><br>Have you have met the above requirement as detailed in our Minimum Standards Reporting Checklist?	

Powered by Editorial Manager® and ProduXion Manager® from Aries Systems Corporation

Manuscript
Click here to access/download;Manuscript;Schneider_et_al_SPRINGENO_11

Two high-quality *de novo* genomes from single ethanol-preserved specimens of tiny metazoans (Collembola).

Clément Schneider[1,2], Christian Woehle[3], Carola Greve[1], Cyrille A. D'Haese[4], Magnus Wolf[1,5,6], Michael Hiller [1,6,7], Axel Janke[1,5,6], Miklós Bálint[1,5,*], Bruno Hüttel[3,*]

[1] LOEWE Centre for Translational Biodiversity Genomics (LOEWE-TBG), Senckenberganlage 25, 60325 Frankfurt am Main, Germany

[2] Senckenberg Gesellschaft für Naturforschung, Abteilung Bodenzoologie, Am Museum 1, 02826 Görlitz, Germany

[3] Max Planck Institute for Plant Breeding Research, Max Planck Genome-centre Cologne, Carl-von-Linné-Weg 10, 50829 Cologne, Germany

[4] Unité Mécanismes adaptatifs & Evolution (MECADEV), CNRS, Muséum national d'Histoire naturelle, 45 rue Buffon 75005 Paris, France.

[5] Senckenberg Biodiversity and Climate Research Centre, Senckenberganlage 25, 60325 Frankfurt am Main, Germany

[6] Goethe University, Max-von-Laue-Str. 9, 60438 Frankfurt am Main

[7] Senckenberg Research Institute, Senckenberganlage 25, 60325 Frankfurt, Germany

[*] these authors contributed equally to the manuscript

Corresponding author:

Clément Schneider

Email: clement.schneider@senckenberg.de

LOEWE Centre for Translational Biodiversity Genomics (LOEWE-TBG), Senckenberganlage 25, 60325 Frankfurt am Main, Germany

Abstract

Background

Genome sequencing of all known eukaryotes on Earth promises unprecedented advances in biological sciences and in biodiversity-related applied fields such as environmental management and natural product research. Advances in long read DNA sequencing make it feasible to generate high-quality genomes for many non-genetic model species. However, long read sequencing today relies on sizable quantities of high-quality, high molecular weight (hmw) DNA which is mostly obtained from fresh tissues. This is a challenge for biodiversity genomics of most metazoan species, which are tiny and need to be preserved immediately after collection. Here we present *de novo* genomes of two species of submillimeter Collembola. For each, we prepared the sequencing library from hmwDNA extracted from a single specimen and using a novel Ultra-Low input protocol from Pacific Bioscience.

Results

The two assembled genomes have N50 values over 5.5 and 8.5 Mb respectively, and both contain ~96% of BUSCO genes. Thus, they are highly contiguous and complete. The genomes are supported by an integrative taxonomy approach including placement in a genome-based phylogeny of Collembola and designation of a neotype for one of the species. Higher heterozygosity values are recorded in the more mobile species. Both species are devoid of the biosynthetic pathway for

beta-lactam antibiotics known in several Collembola, confirming the tight correlation of antibiotics synthesis with the species way of life.

Conclusions

It is now possible to generate high-quality genomes from single-specimens of minute, field-preserved metazoans, exceeding the minimum contig N50 (1Mb) required by the Earth BioGenome Project.

Keywords

Long-read genome sequencing, PacBio, Soil Invertebrates, Eukaryote Biodiversity, Low Input DNA, Integrative Taxonomy

Introduction

Biodiversity genomics employs genome-scale data to study the molecular basis of biodiversity. New genome data and their analyses are currently revolutionizing life- and environmental sciences by addressing scientific questions on evolution, phylogeny, ecology, medicine and other fields of life sciences. One year after the start of the LOEWE Center for Translational Biodiversity Genome (LOEWE-TBG), the Earth BioGenome Project (EBP) announced plans to sequence reference genomes from all known ~1.5 M eukaryotic species [1]. High-quality (highly contiguous and complete, preferentially chromosome-level) genomes sequenced from accurately species-identified organisms are essential for these efforts. To achieve its goal, the biodiversity genomics faces a major challenge: most of the eukaryotic biodiversity belongs to highly diverse families of tiny species [2] that are 1—difficult to sequence and 2—difficult to identify.

Advances in long-read sequencing technology changed the game for biodiversity genomics as this technology now allows to obtain high-quality genomes for diverse taxa. However, minute metazoans pose a number of challenges to long read sequencing. Standard protocols for long-read sequencing require a large input of hmw DNA—in the order of a microgram—which in turn requires larger amounts of fresh or well-preserved input tissue. Pooling individuals from field collected specimens is often not possible and not desirable: many species cannot be captured in sufficiently large numbers, and pooling individuals complicates assembly by increasing genetic heterogeneity and bears the risk of mixing cryptic species. Small animals often need to be preserved as soon as they are removed from their natural habitats. Furthermore, to be precisely identified, individuals have to be sorted, prepared and observed under a microscope. This results in delays between specimen collection and DNA extraction and cannot be done on living specimens. Therefore, most small metazoan species will have to be genome-sequenced from single, field-preserved specimens.

Recent progress has already decreased the amount of DNA needed for long read sequencing. Kingan et al. genome-sequenced a single mosquito on Pacific Biosciences (PacBio) platform [3]. Adams et al. obtained a chromosome level assembly from a single, laboratory bred, fruit fly, based on a combination of Nanopore long reads, Illumina short reads and low input Hi-C sequencing [4]. However, most metazoans are even smaller than a single mosquito or fruit fly and would not yield the amount of DNA required by the applied protocols.

Here we present high-quality genomes of two non-model field collected Collembola species (Arthropoda: Collembola): *Desoria tigrina* (length: 2 mm; Fig. 1A) and *Sminthurides aquaticus* (length: 1 mm; Fig. 1B–E). We extracted DNA from single specimens, preserved for three to 45 days in 96% ethanol, and used a recent whole genome amplification-based Ultra-Low DNA Input

Workflow for SMRT Sequencing (PacBio) [5] to produce libraries from as little as 5 ng DNA input. Using these libraries, we sequenced one SMRT cell for each species. To set the genomes as reliable references, we followed a thorough taxonomic workflow leading to the designation of a needed neotype for *S. aquaticus*. We investigated the resulting genomes for the presence of a beta-lactam antibiotic synthesis pathway, an exceptional trait in the metazoan kingdom known in some species of edaphic Collembola [6]. We placed the two species in a genome-based phylogeny of Collembola. The resulting genomes are highly contiguous and nearly complete. *S. aquaticus* assembly has even the highest contiguity compared to the Collembola genomes sequenced so-far from hundreds of cultured specimens [7,8].

Thus, we show that high-quality, *de novo* genomes can be sequenced following a typical taxonomic workflow, even from submillimeter species that have been preserved for several days in 96 % ethanol. This novel approach will add to the aim of biodiversity genomic to sequence all life on Earth, and make closer the day when whole genome sequencing will be a routine component of integrative taxonomy.

Materials and Methods

Sequenced species

The collembolan *D. tigrina* (Entomobyomorpha, Isotomidae) is a hemidaphous species: it is found in the upper layer of soil and litter. It is mostly found in anthropized environments [9]. It can be very abundant in vegetal compost, is found in crop fields [10], and can occur in caves as a troglophile [11]. In Western Europe it remains active in winter. The collembolan *S. aquaticus* (Symphypleona, Sminthurididae) is an epigeous, hygrophilous species that is widely spread in the

Holarctic region [12]. Specimens often gather plants, wood and rocks emerging from the water surface. The animals can walk and jump on water surfaces thanks to elongated claws and a strong furca (jump appendage) with a tip that functions as a paddle on the surface tension. The species has a remarkably pronounced sexual dimorphism: the male is significantly smaller than the female and its modified antennae into a prehensile organ allows it to clasp the female antenna in a courtship dance preceding external fecundation (Fig. 1 B–E).

Specimens collection and preparation

Desoria tigrina was collected in a garden compost bin (8.5213° E, 50.1393° N, 14.xii.2019). Specimens were extracted from the compost with a Berlese funnel directly into 96% ethanol. DNA extraction was performed within ~72h. *Sminthurides aquaticus* was collected from a pond in a public garden (2.3999° E, 48.8589° N, 27.x.2019). Specimens were caught manually by eye using a small net and mouth-aspirator. They were preserved in 96% ethanol, kept at ambient-temperature for one day until they would be stored at -20°C for 1.5 months, until DNA extraction. Specimens used for morphological identification were cleared in lactic acid and KOH, and they were mounted in permanent slides using Marc-André II mounting medium. Observations were made using a Leitz Wetzlar Diaplan with phase contrast, at 400-1000x magnification.

Ultra-Low Input PacBio sequencing

Extraction was performed from a single specimen for both species. Specimens were rinsed in 1 x PBS (Sigma) to remove residual EtOH. The solution was replaced four times with fresh PBS. Specimens were crushed by one-way pistils (Sigma) then DNA was extracted using the Qiagen

MagAttract kit (Hilden, Germany). DNA was eluted once in 40 µl AE buffer. We performed eight individual extractions from *S. aquaticus* and four individual extractions from *D. tigrina* specimens. Each DNA extract was quantified with the Quantus dsDNA system (Promega) and DNA quality was assessed with FEMTOpulse (Agilent). One DNA extract was selected for each species (FEMTOpulse measurements are provided in supplementary file S1).

Libraries were prepared using an early access kit for the Ultra-Low DNA Input Workflow for SMRT Sequencing (PacBio) [5], that was kindly provided by PacBio. Total input DNA was approximately 59.24 ng (*D. tigrina*) and 16.16 ng (*S. aquaticus*), respectively. Of the genomic hmwDNA extracts, 5 ng was fragmented with g-Tubes (Covaris). The resulting fragment sizes were again inspected with FEMTOpulse (Agilent). Next, single-stranded overhangs were enzymatically removed, followed by DNA damage repair, repair of DNA ends, and an A-tailing step. Double-stranded DNA adapter with a T-overhang was ligated for 1 h at 20°C and the resulting products were bead purified (ProNex, Promega), eluted and split into two identical aliquots. DNA fragments with adapters were amplified in two different PCR reactions (reaction 1: 98°C for 45 s, 14 cycles: 98°C for 10s, 62°C for 15s, 72°C for 7 min, final elongation 72°C 5 min; reaction 2: 98°C for 30 s, 14 cycles: 98°C for 10s, 60°C for 15s, 68°C for 10 min, final elongation 68°C 5 min). PCR reactions were again bead purified, and eluted in EB. Library fragments were assessed for quantity (Quantus, Promega) and quality (FEMTOpulse, Agilent). PCR fragments from both reactions were pooled in equal concentrations to achieve a total of 500 ng input for library preparation. Libraries were prepared following the Low DNA Input Workflow for SMRT Sequencing (PacBio, California). Libraries were annealed to a sequencing primer (V4), bound to Sequel II DNA polymerase 2.0 with Binding kit 2.0 and sequenced in a Sequel II 8M SMRT cell for 30 h.

Genome assembly

Generation of circular consensus sequencing (CCS) reads and adapter trimming was done in PacBio SMRTLink 8 with default parameters followed by deduplication of reads via pbmarkdup (v0.2.0 [13]) as recommended by PacBio. HiFi reads containing complete PCR adapter sequences were discarded. Genome properties were estimated with kmer statistics prior to assembly. This is possible due to the low error rates of HiFi reads. K-mers were counted and aggregated using jellyfish 2.2.10 [14] ('jellyfish count -C -m 21 -t 20 -s 1000000000 -o jelly_k21.jf CCS.fasta' & 'jellyfish histo -t 10 jelly_k21.jf > kmer.histo'). We used GenomeScope 1.0 [15] to estimate genome length, level of duplication and heterozygosity through the web application [16].

Several long read assembly tools were compared: FALCON (falcon-kit v1.8.0) [17], Flye (v2.9.1-b1676) [18], HiCanu (v2.1) [19], Hifiasm (v0.12-r304) [20], IPA (v1.1.2) [21] and wtdbg2 (v2.5) [22]. The command lines and statistics of preliminary assemblies are provided in supplementary file S2. We retained Hifiasm, which produced the assemblies with significantly higher N50 for both species. We repeated the assembly process with Hifiasm after removing 5% and 10% of the shortest reads. Further separation of haplotigs was performed using alternatively purge_dups [23] (v1.0.1) or purge_haplotigs [24] (v1.1.0), and for each species we retained the method that achieved better Benchmarking Universal Single-Copy Orthologs (BUSCO) deduplication. The effect of purging on genome completeness was assessed with BUSCO v4.1.4 [25], in genome mode and with the 'long' option, with the arthropoda_odb10 dataset [26]. For each species, we selected the method that led to the highest N50 and optimal purging (lowest amount of duplicated BUSCOs without significant loss of complete BUSCOs). For additional polishing of the resulting assemblies, we followed PacBio guidelines [17]. We used racon [27] (v1.4.10, parameter: '-u') in combination

with samtools [28] (V1.9, parameters: 'view -F 1796 -q 20') and pbmm2 [29] (v1.1.0, parameters: '--preset CCS --sort'), a wrapper of minimap2 [30].

To assemble the mitochondrial genomes, we gathered CCS containing exclusively mitochondrial sequences used blastn (blastn+ suite v2.10.0, [31]) and assembled them using Geneious 2020.1.2 [32]. Circularity was validated manually, and nucleotide bases were called with a 75% threshold consensus. The mitochondrial genomes were annotated with the MITOS2 web server [33]. Coding DNA sequences were checked and corrected using Geneious, to ensure that the presence of uncommon start codon and incomplete stop codons did not mislead the automatic annotation algorithms. Boundaries of the rDNAs were slightly adjusted to make them contiguous with the tRNA(val) gene.

We used blastn to identify insertions of the mitochondrial genome in the nuclear genome (NUMTs). For this query, we used a 2x duplicated sequence of the mitochondrial genome to handle circularity. We recognized the presence of almost complete copies of the mitochondrial genome in the nuclear genomes of both species. We investigated the mapping of the CCS to the assembly in those locations using IGV [34] (v2.8.13), and recognized that in one instance, a mis-assembly occurred through the soldering of two NUMTs with CCS of mitochondrial origin. All CCS aligning with those two NUMTs were gathered with blastn and reassembled using Geneious. We could not find unambiguous NUMTs CCS (i.e. CCS carrying both nuclear and mitochondrial sequence) that would support the original assembly connection and therefore we split the contig.

Contamination control

We checked the assemblies for potential contamination from other organisms by querying the contigs against the National Center for Biotechnology Information (NCBI) database using protein-

based (DIAMOND, [35]) and nucleotide-based (blastn) alignments. Results were merged with Blobtools2 [36] (v2.3.3) using the "bestsum" algorithm. Contigs explicitly assigned to another lineage than metazoan were excluded from the assembly. Contigs assigned to Chordata were checked for presence of Arthropoda BUSCO. If Arthropoda BUSCOs were confirmed on such contigs, we retained them for the assembly.

Assembly assessment

Curated assemblies were again evaluated with BUSCO (same parameters as before). We mapped the CCS on the assemblies using backmap [37] (v0.3), a perl wrapper of minimap2 and QualiMap2 [38]. Minimap2 was run with "-H -ax asm10" to map CCS on the assembly. We then performed another estimation of the genome size by dividing the number of mapped nucleotides by mode of the coverage distribution [37].

Comparison with previous long read assemblies

We compared our new genomes sequenced to previous Collembola assemblies that were generated with long read and sometimes additional short read data. We also compared our Collembola assemblies to genomes of two larger insects [3,39] (4 and 20 mm), which were also sequenced from single specimens but with the PacBio Low input workflow [40] (amplification-free).

Genome annotation

The assemblies were annotated with *ab initio* gene prediction. Repetitive regions were masked with RepeatModeler [41] (v2.0.1) with the options: '-LTRStruct -engine ncbi' using RepeatMasker [42] (open-4.0.9, options: '-xsmall -gff -nolow'). Protein sequences were predicted with AUGUSTUS [43] (v3.3.3, option: '--softmasking=on') re-using the BUSCO training results. Functional

annotations were obtained by a local installation of eggNOG-mapper [44] (v2.0.1, option: '-m diamond‘). If emapper recovered no annotations, we denoted sequences as 'hypothetical protein' (for proteins without hits in emapper), or 'uncharacterized protein' (for proteins with hits without annotations). To determine if *D. tigrina* and *S. aquaticus* share the beta-lactams synthesis gene found in some other Collembola, we searched the genomes for genes homologous of the isopenicillin N synthase (IPNS) and δ-(L-α-aminoadipoyl)-L-cysteine-D-valine synthetase (ACVS) genes of *Folsomia candida*. Those two genes belong to the same gene cluster. We used blastn: blastn and megablast to query the DNA sequences against the genome assembly and blastp to query the protein sequences against the predicted proteins sequences from the genomes. The NBCI accession number of the searched sequences are: IPNS—JX270832.1, ACVS—OXA60265.1.

Phylogenetic analysis

We gathered 13 Collembola genome assemblies [7,8,45,46] from NCBI. For the outgroup, we selected a Diplura [47] and a Diptera [3] genome assemblies. The species list and the genomes accession numbers are provided in Table 1. We used BUSCO v4.0.6 in short mode to search for orthologs, restricting the search to the arthropoda_odb10 dataset. We screened the obtained BUSCO sets to identify genes shared among the species, allowing only genes found for at least 75 % of the species. We aligned single protein sequences with MAFFT [48] (v7.450), concatenated the alignments with FASconCAT-G [49] (v1.04), and trimmed the final alignment with trimAl [50] (v1.2). We calculated a maximum likelihood tree with IQtree [51] (v1.6.12) with 1000 non-parametric bootstrap replications.

Results

Species biology and taxonomy

In terms of biomass and number *D. tigrina* was by far the dominant Collembola found in the compost bin during the winter season Morphological observations placed the collected specimens unambiguously within the *D. tigrina*-group [52]. Within this group, outer maxillary palp chaetotaxy was used to distinguish *D. tigrina* from its sibling species *D. grisea* following Fjellberg [52]. Identification was further validated following Potapov [9]. Six females, two males and two juveniles on four slides numbered EA013940-43 were deposited in the Apterygota collection of the National Museum of Natural History, Paris. Seventeen females and three males on 12 slides labelled CSCH-1326—1337 were deposited in the Apterygota collection at Senckenberg, Görlitz. *Sminthurides aquaticus* was the only Collembola forming a population on the pond at the site of collection (i.e. no accidental fall on water surface). Abundantly found in October 2019, it was observed again in June 2020 in large numbers and with courtship behavior undergoing (Fig 1D, E). The specimens identification was unambiguous following [12,52,53]. One male on a slide numbered CSCH-1338 is designated as the neotype for *S. aquaticus* (see discussion) and will be deposited in the Apterygota collection at the National Museum of Natural History, Paris, along with three females and two males on five slides (CSCH-1339–1344) and 20 individuals in 96% ethanol (CS.371, leg. C. Schneider). Three males, three females and one juvenile on five slides numbered CSCH-1345–1349 were deposited in the Apterygota collection at Senckenberg, Görlitz.

DNA sequencing

For *D. tigrina* a total of 20,22 Gb HiFi data (Q>=20) was generated, with mean read length of 12,155 bp, median read length of 11,792 bp and max read length of 37,982 bp. The distribution of read length is reported in Fig. 2. From the kmer content of the reads, the genome haploid length was estimated to be ca 168 Mb with 1.43 % of heterozygosity and 3 % duplications.

For *S. aquaticus* a total of Gb HiFi data (Q>=20) was generated with mean length of 12,308 bp, median read length of 11,893 bp and max read length of 29,073 bp. The distribution of read length is reported in Fig. 2. From the kmer content of the reads, the genome haploid length was estimated to be ca 152 Mb with 0.96 % of heterozygosity and 0.78% duplications.

Genome assembly

Overall, Hifiasm produced the best assemblies for both species (supplementary file S2).

For *D. tigrina*, the most contiguous assembly (Table 2, Fig. 3) was obtained by selecting 95 % of the reads excluding the shortest one. Purging haplotigs with Purge_dups resulted in less duplicated BUSCOs than with Purge_haplotigs. No contigs were found to be of non-metazoan origin. While some contigs were assigned to Chordata taxa, they all carried Arthropoda specific BUSCOs and were therefore kept. The curated primary assembly of *D. tigrina* is composed of 142 contigs, has a size of 211,462,971 bp and an N50 value of 5.63 Mb (Table 2, Fig. 3). Mean coverage is 95.40 X with a coverage distribution mode of 103 X. The genome size is 196 Mbp, estimated from mapped reads and coverage. BUSCO search on the whole assembly yielded 96 % complete BUSCOs (including 1.7 % duplicated), 0.9 % fragmented BUSCOs and 3.1 % missing BUSCOs. The mitochondrial genome assembly was complete, for a size of 15,139 bp. Two large NUMTs were

found, each on a different contigs. One was 18,113 bp (120 % of the mitochondrial genome size), the other one was 28,173 bp (186 % of the mitochondrial genome size). Examination of the mapped reads revealed no obvious mis-assembly for the smaller NUMT (spanned by reads that contained mitochondrial and genomic sequence), but the larger NUMT was bridged in the middle by reads containing exclusively mitochondrial. Therefore, we split the contig carrying the larger NUMT, keeping on each side a partial NUMT sequence supported by reads containing mitochondrial and genomic sequence.

For *S. aquaticus* the best assembly was obtained by using all the reads (Table 2, Fig. 3). Purging haplotigs with Purge_haplotigs resulted in less duplicated BUSCOs than with Purge_dups. Two contigs (totalizing 243,436 bp) were found to be from a fungi and a cyanobacteria respectively, and were removed. Some contigs were assigned to Chordata taxa but all of those carried Arthropoda specific BUSCOs and were kept. The curated assembly of *S. aquaticus* is composed of 79 contigs, has a size of 165,915,169 bp and an N50 value of 8.78 Mb (Table 2, Fig. 3). Mean coverage is 72.67 X, coverage distribution mode is 77 X. The genome size is 157 Mb, estimated from mapped reads and coverage. BUSCO search on the whole assembly yielded 96.1 % complete BUSCOs (including 1.6 % duplicated), 1.3 % fragmented BUSCOs and 2.6 % missing BUSCOs. The mitochondrial genome assembly was complete, for a size of 16,099 bp. A large NUMT was detected in one of the purged contigs (haplotigs), but none were found in the primary contigs, so we decided to not investigate further. Several small contigs were found to be assembled from mitochondrial reads and were removed.

Comparison with previous long read assemblies

In terms of BUSCO completeness scores, our assemblies are comparable to previous high-quality Collembola genomes assembled from a large pool of specimens (95.8% and 96.1% vs. 94.5 –97.1% complete; Table 2). In terms of assembly contiguity, our *S. aquaticus* has the highest and the *D. tigrina* assembly has the third-highest contig N50 value (Table 2, Fig. 3). The insect genomes have higher BUSCO scores (96.5 and 99.6%), but lower contiguity (Table 2, Fig. 3). Together, this shows that assemblies generated with the Ultra-Low input workflow and long read sequencing can reach or surpass the level of quality of assemblies obtained with the standard or Low-Input workflow.

Genome annotation

In the mitochondrial genome of both species, we identified the complete set of 37 mitochondrial genes (13 proteins, 22 tRNA and 2 rRNA coding genes) typically found in Hexapoda. In the nuclear genome, we predicted 24,423 proteins for *D. tigrina*, 15,546 (63.65%) of which had homologs in other organisms and 8,877 were labeled as "hypothetical protein". BUSCO search on the predicted proteins yielded 96.2% complete BUSCO including 2.6% duplicated, 1.2% fragmented and 2.6% missing. For *S. aquaticus*, we predicted 17,624 proteins in the nuclear genome, 11,989 (68.03%) of which had homologs in other organisms and 5,635 were labeled "hypothetical protein". BUSCO search on the predicted proteins yielded 95.3% complete BUSCO including 2.1% duplicated, 1.6% fragmented and 3.1% missing.

Beta-lactam biosynthetic pathway

Collembola exhibit a diversity in the presence of a beta-lactam antibiotic synthesis pathway which is secondarily lost in some species. Therefore, we analyzed our genomes for the presence of a beta-lactam antibiotic synthesis pathway. No homologs of the genes IPNS and ACVS could be identified in the two genomes, indicating the absence of the beta-lactam antibiotic synthesis pathway in *D. tigrina* and *S. aquaticus*. However, by screening the functional annotation of the predicted genes, we identified four genes related to aminopenicillanic-acid-acyltransferase (penDE) in the genome of *S. aquaticus* and five penDE-like genes in the genome of *D. tigrina*.

Phylogeny

To place our two species in a molecular phylogeny of Collembola, we used BUSCO genes as conserved phylogenetic markers. Allowing for a maximum of 25% missing sequence for each ortholog, we retained 545 complete BUSCOs to align. The total length of the trimmed alignments is 171,703 sites. We used IQTree to infer a phylogenetic tree, shown in Fig. 4. Our two newly sequenced species find their expected placement on the Collembola phylogeny with *Sminthurides aquaticus* as a sister species to *S. bifidus* (both are representants of the genus *Sminthurides*, family Sminthurididae) and *D. tigrina* as a sister species to *F. candida* (both are representants of the family Isotomidae). Our tree also recovered the monophyly of orders Symphypleona, Poduromorpha and Entomobryomorpoha with 100 % bootstrap support. However, the basal relationships between the four orders of Collembola receive negligible bootstrap support (= 73%), indicating phylogenetic irresolution. The rest of the tree is consistent with the to-date most detailed genome-based phylogeny of Sun et al. [46]. The more than 400 million years old basal relationships of Collembola

are long debated. They are sensitive to data sampling, and phylogenetic artifacts such as long branch attraction and random root occur [54]. Additional genomes of key Collembola representatives and more informative phylogenetic markers [46] are needed to properly address the problem of basal relationships within Collembola.

Discussion

Value of the Ultra-Low input workflow

Long read sequencing as the future for *de novo* genome assembly normally required larger amount of input tissue, which limits its application to larger organisms. However, a substantial portion of biodiversity is represented by tiny species. Here, we address this important challenge in biodiversity genomics and provide a proof of concept that it is now possible to sequence high quality reference genomes from field collected individual tiny Collembola species. The 5 ng input of the PacBio Ultra-Low Input Workflow is a significant drop from the 150 ng input required by the PacBio Low Input Workflow (WGA-free). And yet the ultra-low input still allow to capture high-quality genomic data: our final assemblies were of high contiguity and completeness on par with recent genomes from larger insects sequenced using the low input protocol [3,39]. Our new genomes are also on par with the previously best reference genomes for Collembola; *F. candida* and *Sinella curviseta*, which were DNA sequenced from hundreds of specimens maintained in culture [7,8]. *Sminthurides aquaticus* even achieve the highest N50 and N75 among the compared assemblies. The quality of the new assemblies makes us consider that there are even further benefits in the ultra-low input protocol than sequencing organisms too small for WGA-free approaches. For not too small species, it can be used to generate long reads data from a fraction of the total DNA.

This could be levered to implement approaches combining long-read and Hi-C for even smaller species than a fruit fly [4]. This can also allow to retain the sequenced specimen to serve as a voucher, by removing the need to crush the specimen to maximize hmwDNA recovery.

Ensuring taxonomic quality

It is essential that a reliable reference genome is supported by a solid and revisable taxonomy, to be useful for any meaningful downstream analysis. Taxonomy quality has always been an issue of sequence databases [55,56]. This is especially true for field collected specimens from taxonomically poorly known groups that are often riddled with cryptic diversity and difficulty of species identification based on a few subtle characters. Therefore, we documented species collection and identification by morphological characters, provided macro-photographs, and preserving co-captured specimens of the same species in the collection of two European museums. This way, we ensure the taxonomic traceability of the reference genome, which should be a prerequisite for any meaningful biodiversity genomics where species identification is not straight forward.

The genus *Desoria* has a complex taxonomy. Within the *D. tigrina* group sensu Fjellberg 2007 [52], *D. tigrina* and *D. grisea* are two sibling species, described in the early times of modern Collembola systematics. *Desoria grisea* was redescribed by Fjellberg [52] from its type locality. Fjellberg reported that the two species, while extremely similar, could be consistently distinguished by the organization of the labial palp chaetae. We examined several specimens from our collection spot and each of them were identified as *D. tigrina*, supporting the identity of the specimen used for sequencing.

Sminthurides aquaticus was originally described from France and has been recognized to be widely spread throughout the Holarctic region. We confirmed that all our collected specimens are identical to the accepted descriptions of *S. aquaticus*. The species was originally described by Bourlet in 1841 probably from the north of France. However, Bourlet did not make any reference to a type series, and to our knowledge did not preserve any specimens. We consider that the population we sampled in Paris is suitable to provide a neotype for this species: the population is abundant, settled, and easily accessible for further studies. This also offers the uncommon opportunity to have a neotype closely related to the reference genome for the species.

Heterozygosity

The higher level of heterozygosity in *D. tigrina* compared to *S. aquaticus* seems consistent with the expected level of isolation of the populations. *D. tigrina* invaded the compost that was set up one year before the collection. The species is very mobile, being rather large and equipped with a long furca, and gene flow must be active across the nearby surrounding fields and gardens. On the other hand, the sampled population of *S. aquaticus* seems rather isolated in a small area (artificial pond in a public garden).

Beta-lactam synthesis in Collembola.

Recent results from transcriptomes show that several edaphic species from the orders Poduromorpha and Entomobryomorpha can synthesize beta-lactam antibiotics [6]. Two essential genes of the beta-lactam synthesis pathway, ACVS and IPNS, are consistently found in four euedaphic species (“true” soil dweller), but missing in two out of four hemidaphic species (living

in upper layer of soil, litters and dead wood), and always missing in seven atmobiotic species (species living on vegetation, fresh water surface or tidal zone). The genes are absent from soil dwellers from the class Diplura and Protura, two close relatives of Collembola. The antibiotic biosynthesis likely resulted from a single horizontal gene transfer event with subsequent loss of antibiotic synthesis ability in some of the investigated species [6].

We report the absence of the ACVS and IPNS in the genomes of *S. aquaticus* and *D. tigrina*. *Sminthurides aquaticus* belongs to a family of Symphypleona which was not investigated by Suring et al [6]. So far, no Symphypleona are known to carry those genes, but it must be noted that none of the tested species are soil dwelling species. The Symphypleona species in Suring et al. [6] dataset are vegetation dwellers. Since *S. aquaticus* dwells on fresh water surfaces, our results support the lack of antibiotic production in semi-aquatic species. The absence of the genes in *D. tigrina* is rather unexpected, since the species lives in organic-rich litter with potentially high microbial contents. After *F. candida*, *D. tigrina* is the second member of the large Isotomidae family to be investigated for antibiotic production. *Desoria tigrina* is in the same class size as *F. candida*, but is expected to be more mobile due to its more developed legs, furca and eye-patch (*F. candida* is eyeless). This suggests that antibiotic synthesis is specific to true soil dwelling (euedaphic) life-style, and it might be lost by more mobile species.

Antibiotic synthesis in Collembola

Both *D. tigrina* and *S. aquaticus* possess penDE-like genes. Such genes were also reported in *F. candida* [6]. The penDE is the last enzyme in the penicillin biosynthetic pathway of the fungi *Emericella nidulans*, and converts isopenicillin N (product of INPS activity) to penicillin G. In F. candida, the penDE-like gene does not belong to the beta-lactam synthesis gene cluster. Homologs

of penDE are also known in fungi that do not produce antibiotics. Suring et al. [6] suggest that penDE-like genes may have been co-opted for the completion of the penicillin synthesis in *F. candida* after the acquisition of the beta-lactam synthesis gene cluster. Consequently, the presence of penDE in *S. aquaticus*, and *D. tigrina* is not a solid indicator of a lost antibiotic production trait in these species. Altogether, the assumption that the horizontal gene transfer is an ancestral acquisition to Collembola should be taken with caution since the basal relationships between Collembola orders are still unresolved. For further elucidation, edaphic species of orders Symphypleona and Neelipleona should be investigated for the antibiotic production trait.

Conclusions

The LOEWE-TBG excellence cluster supports the idea of the EBP that aims to sequence all eukaryotic species. Although the first high-quality genomes were generated for species with easy access to abundant and fresh samples, similar high-quality genomes can now be generated for tiny taxa or taxa that is otherwise difficult to sequence. Most of known eukaryotic biodiversity belongs to very small metazoan which in addition needs to be preserved for some time before genome sequencing. Access to their genomes provides insights into the formation, maintenance and functioning of eukaryotic biodiversity, and presents new opportunities for natural resource management and bioprospecting. The ability to genome-sequence these species is essential for the success of biodiversity genomics initiatives. Our genomes sequenced from 5 ng DNA actually exceed the 1Mb N50 contig continuity required by the EBP project when more than 100 ng DNA are available. We are convinced that integrating high-quality genomics with the typical workflow of small, field-collected metazoans is an essential approach toward the creation of a solid reference

genomes database for millions of minute non-model species belonging to taxonomically challenging groups.

Data Availability

The project is deposited in the EMBL-ENA database under accession number PRJEB39696 including: *S. aquaticus* CCS, curated assembly and annotation under accessions numbers ERR4407379, ERZ1473260, ERZ1473263, *D. tigrina* CCS, curated assembly and annotation under accession numbers ERR4407422, ERZ1473259, ERZ1473262.

Supporting data, including assembly and annotation files, will also be made available via the *GigaScience* database, GigaDB.

Additional Files

Supplementary file S1. Report on preliminary assemblies, including assembly statistics and details of assembly tools and command lines.

Abbreviations

bp: base pairs; BUSCO: Benchmarking Universal Single-Copy Orthologs; Gb: gigabase pairs; hmw: high molecular weight; kb: kilobase pairs; LOEWE-TBG: LOEWE Center for Translational Biodiversity Genomics; Mb: megabase pairs; NCBI: National Center for Biotechnology Information; PacBio: Pacific Biosciences.

Competing Interests

The authors declare that they have no competing interests.

Authors' Contributions

C.S. conceived the project; C.S. and C.D'H. collected, identified and photographed the specimens; B.H. performed the DNA extraction, the library preparation and the sequencing; C.W. and C.S. assembled and analyzed the genomes; M.W. and A.J. contributed the phylogenomic analysis; C.S., M.B. led the writing of the manuscript; C.G. performed experiments (not presented here) that helped steering the project and further advised on the study; M.H. revised the manuscript. All authors read and approved the final manuscript for submission.

ACKNOWLEDGEMENTS

The present study is a collaboration between of the LOEWE-TBG and the Max Planck Genome-centre Cologne. It was supported through the programme "LOEWE – Landes-Offensive zur Entwicklung Wissenschaftlich-ökonomischer Exzellenz" of Hesse's Ministry of Higher Education, Research, and the Arts. We highly appreciate the generous support by Pacific Bioscience with respect to the ultra-low amplification kit, library preparation kit as well as SMRT cells and sequencing chemistry during the course of the beta test. The Max-Planck Genome Center Cologne acknowledges the support from the Max-Planck Society. We give our warm thanks to Tilman Schell for his advice on genome assembly.

References

1. Lewin HA, Robinson GE, Kress WJ, Baker WJ, Coddington J, Crandall KA, et al. Earth BioGenome Project: Sequencing life for the future of life. *Proc Natl Acad Sci USA*. 2018; doi: 10.1073/pnas.1720115115.

2. Stork NE, McBroom J, Gely C, Hamilton AJ. New approaches narrow global species estimates for beetles, insects, and terrestrial arthropods. *Proc Natl Acad Sci USA*. 2015; doi: 10.1073/pnas.1502408112.

3. Kingan SB, Heaton H, Cudini J, Lambert CC, Baybayan P, Galvin BD, et al. A High-Quality De novo Genome Assembly from a Single Mosquito Using PacBio Sequencing. *Genes (Basel)*. 2019; doi: 10.3390/genes10010062.

4. Adams M, McBroome J, Maurer N, Pepper-Tunick E, Saremi NF, Green RE, et al. One fly–one genome: chromosome-scale genome assembly of a single outbred Drosophila melanogaster. *Nucleic Acids Res*. Oxford Academic; 2020; doi: 10.1093/nar/gkaa450.

5. PacBio: Now Available: Ultra-Low DNA Input Workflow for SMRT Sequencing. PacBio. https://www.pacb.com/blog/introducing-the-ultra-low-input-protocol-for-smrt-sequencing/ (2020). Accessed 2020 Dec 4.

6. Suring W, Meusemann K, Blanke A, Mariën J, Schol T, Agamennone V, et al. Evolutionary ecology of beta-lactam gene clusters in animals. *Molecular Ecology*. 2017; doi: 10.1111/mec.14109.

7. Faddeeva-Vakhrusheva A, Kraaijeveld K, Derks MFL, Anvar SY, Agamennone V, Suring W, et al. Coping with living in the soil: the genome of the parthenogenetic springtail Folsomia candida. *BMC Genomics*. 2017; doi: 10.1186/s12864-017-3852-x.

8. Zhang F, Ding Y, Zhou Q-S, Wu J, Luo A, Zhu C-D. A High-quality Draft Genome Assembly of Sinella curviseta: A Soil Model Organism (Collembola). *Genome Biol Evol*. Oxford Academic; 2019; doi: 10.1093/gbe/evz013.

9. Potapov, M. Synopses on Palaearctic Collembola, Volume 3, Isotomidae. *Abhandlungen und Berichte des Naturkundemuseums, Görlitz*. 73:1–6032001;

10. Gruss I, Twardowski J. The assemblages of soil-dwelling springtails (Collembola) in winter rye under long-term monoculture and crop rotation. *Zemdirbyste-Agriculture*. 2016; doi: 10.13080/z-a.2016.103.021.

11. Dányi L. Cave dwelling springtails (Collembola) of Hungary: a review. *Soil Organisms*. 83:419–322011;

12. Bretfeld G. Synopses on Palaearctic Collembola : Symphypleona. *Abhandlungen und Berichte des Naturkundemuseums Gorlitz*. 71:1–3181999;

13. PacBio: PacificBiosciences/pbmarkdup. https://github.com/PacificBiosciences/pbmarkdup (2020). Accessed 2020 Mar 1.

14. Marçais G, Kingsford C. A fast, lock-free approach for efficient parallel counting of occurrences of k-mers. *Bioinformatics*. 2011; doi: 10.1093/bioinformatics/btr011.

15. Vurture GW, Sedlazeck FJ, Nattestad M, Underwood CJ, Fang H, Gurtowski J, et al. GenomeScope: fast reference-free genome profiling from short reads. *Bioinformatics*. 2017; doi: 10.1093/bioinformatics/btx153.

16. : Cold Spring Harbor Laboratory: GenomeScope. http://qb.cshl.edu/genomescope. Accessed 2020 Apr 15.

17. PacBio: PacificBiosciences/pbbioconda. GitHub. https://github.com/PacificBiosciences/pbbioconda. Accessed 2020 Mar 1.

18. Kolmogorov M, Yuan J, Lin Y, Pevzner PA. Assembly of long, error-prone reads using repeat graphs. *Nat Biotechnol*. 2019; doi: 10.1038/s41587-019-0072-8.

19. Nurk S, Walenz BP, Rhie A, Vollger MR, Logsdon GA, Grothe R, et al. HiCanu: accurate assembly of segmental duplications, satellites, and allelic variants from high-fidelity long reads. *Genome Res*. 2020; doi: 10.1101/gr.263566.120.

20. Cheng H, Concepcion GT, Feng X, Zhang H, Li H. Haplotype-resolved de novo assembly with phased assembly graphs. *arXiv:200801237 [q-bio]*. 2020;

21. PacBio : PacificBiosciences/pbipa. https://github.com/PacificBiosciences/pbipa (2020). Accessed 2020 Sep 12.

22. Ruan J, Li H. Fast and accurate long-read assembly with wtdbg2. *Nature Methods*. Nature Publishing Group; 2020; doi: 10.1038/s41592-019-0669-3.

23. Guan D, McCarthy SA, Wood J, Howe K, Wang Y, Durbin R. Identifying and removing haplotypic duplication in primary genome assemblies. *Bioinformatics*. Oxford Academic; 2020; doi: 10.1093/bioinformatics/btaa025.

24. Roach MJ, Schmidt SA, Borneman AR. Purge Haplotigs: allelic contig reassignment for third-gen diploid genome assemblies. *BMC Bioinformatics*. 2018; doi: 10.1186/s12859-018-2485-7.

25. Seppey M, Manni M, Zdobnov EM. BUSCO: Assessing Genome Assembly and Annotation Completeness. In: Kollmar M, editor. *Gene Prediction: Methods and Protocols*. New York, NY: Springer;

26. Kriventseva EV, Kuznetsov D, Tegenfeldt F, Manni M, Dias R, Simão FA, et al. OrthoDB v10: sampling the diversity of animal, plant, fungal, protist, bacterial and viral genomes for evolutionary and functional annotations of orthologs. *Nucleic Acids Res*. Oxford Academic; 2019; doi: 10.1093/nar/gky1053.

27. Sovic I: isovic/racon. https://github.com/isovic/racon (2020). Accessed 2020 Mar 2.

28. : Samtools. http://www.htslib.org/ Accessed 2020 Mar 2.

29. PacBio : PacificBiosciences/pbmm2. https://github.com/PacificBiosciences/pbmm2 (2020). Accessed 2020 Mar 12.

30. Li H. Minimap2: pairwise alignment for nucleotide sequences. *Bioinformatics*. Oxford Academic; 2018; doi: 10.1093/bioinformatics/bty191.

31. Camacho C, Coulouris G, Avagyan V, Ma N, Papadopoulos J, Bealer K, et al. BLAST+: architecture and applications. *BMC Bioinformatics*. 2009; doi: 10.1186/1471-2105-10-421.

32. Geneious : Geneious | Bioinformatics Software for Sequence Data Analysis. https://www.geneious.com/ Accessed 2020 Dec 2.

33. Bernt M, Donath A, Jühling F, Externbrink F, Florentz C, Fritzsch G, et al. MITOS: Improved de novo metazoan mitochondrial genome annotation. *Molecular Phylogenetics and Evolution*. 2013; doi: 10.1016/j.ympev.2012.08.023.

34. Robinson JT, Thorvaldsdóttir H, Winckler W, Guttman M, Lander ES, Getz G, et al. Integrative genomics viewer. *Nature Biotechnology*. Nature Publishing Group; 2011; doi: 10.1038/nbt.1754.

35. Buchfink B, Xie C, Huson DH. Fast and sensitive protein alignment using DIAMOND. *Nature Methods*. Nature Publishing Group; 2015; doi: 10.1038/nmeth.3176.

36. Challis R, Richards E, Rajan J, Cochrane G, Blaxter M. BlobToolKit – Interactive Quality Assessment of Genome Assemblies. *G3: Genes, Genomes, Genetics*. G3: Genes, Genomes, Genetics; 2020; doi: 10.1534/g3.119.400908.

37. Schell T, Feldmeyer B, Schmidt H, Greshake B, Tills O, Truebano M, et al. An Annotated Draft Genome for Radix auricularia (Gastropoda, Mollusca). *Genome Biology and Evolution*. 2017; doi: 10.1093/gbe/evx032.

38. Okonechnikov K, Conesa A, García-Alcalde F. Qualimap 2: advanced multi-sample quality control for high-throughput sequencing data. *Bioinformatics*. Oxford Academic; 2016; doi: 10.1093/bioinformatics/btv566.

39. Kingan SB, Urban J, Lambert CC, Baybayan P, Childers AK, Coates B, et al. A high-quality genome assembly from a single, field-collected spotted lanternfly (Lycorma delicatula) using the PacBio Sequel II system. *Gigascience*. Oxford Academic; 2019; doi: 10.1093/gigascience/giz122.

40. Duncan, Turner, Sarah B. Kingan, Christine C. Lambert, Primo Baybayan, and Jonas Korlach. "A Low DNA Input Protocol for High-Quality PacBio De Novo Genome Assemblies." *Journal of Biomolecular Techniques : JBT* 30, no. Suppl (December 2019): S1–2.

41. Flynn JM, Hubley R, Goubert C, Rosen J, Clark AG, Feschotte C, et al. RepeatModeler2 for automated genomic discovery of transposable element families. *PNAS*. National Academy of Sciences; 2020; doi: 10.1073/pnas.1921046117.

42. Smit A, Hubley R, Green P: RepeatMasker Open-4.0. http://www.repeatmasker.org Accessed 2020 Sep 12.

43. Stanke M, Keller O, Gunduz I, Hayes A, Waack S, Morgenstern B. AUGUSTUS: ab initio prediction of alternative transcripts. *Nucleic Acids Res*. Oxford Academic; 2006; doi: 10.1093/nar/gkl200.

44. Huerta-Cepas J, Szklarczyk D, Heller D, Hernández-Plaza A, Forslund SK, Cook H, et al. eggNOG 5.0: a hierarchical, functionally and phylogenetically annotated orthology resource based on 5090 organisms and 2502 viruses. *Nucleic Acids Res*. Oxford Academic; 2019; doi: 10.1093/nar/gky1085.

45. Faddeeva-Vakhrusheva A, Derks MFL, Anvar SY, Agamennone V, Suring W, Smit S, et al. Gene Family Evolution Reflects Adaptation to Soil Environmental Stressors in the Genome of the Collembolan Orchesella cincta. *Genome Biol Evol*. 2016; doi: 10.1093/gbe/evw134.

46. Sun X, Ding Y, Orr MC, Zhang F. Streamlining universal single- copy orthologue and ultraconserved element design: A case study in Collembola. *Mol Ecol Resour*. 2020; doi: 10.1111/1755-0998.13146.

47. i5K Consortium. The i5K Initiative: advancing arthropod genomics for knowledge, human health, agriculture, and the environment. *J Hered*. 2013; doi: 10.1093/jhered/est050.

48. Katoh K, Standley DM. MAFFT Multiple Sequence Alignment Software Version 7: Improvements in Performance and Usability. *Mol Biol Evol*. Oxford Academic; 2013; doi: 10.1093/molbev/mst010.

49. Kück P, Longo GC. FASconCAT-G: extensive functions for multiple sequence alignment preparations concerning phylogenetic studies. *Front Zool*. 2014; doi: 10.1186/s12983-014-0081-x.

50. Capella-Gutiérrez S, Silla-Martínez JM, Gabaldón T. trimAl: a tool for automated alignment trimming in large-scale phylogenetic analyses. *Bioinformatics*. Oxford Academic; 2009; doi: 10.1093/bioinformatics/btp348.

51. Nguyen L-T, Schmidt HA, von Haeseler A, Minh BQ. IQ-TREE: A Fast and Effective Stochastic Algorithm for Estimating Maximum-Likelihood Phylogenies. *Mol Biol Evol*. Oxford Academic; 2015; doi: 10.1093/molbev/msu300.

52. Fjellberg A. The Collembola of Fennoscandia and Denmark Part II : Entomobryomopha and Symphypleona. *Fauna Entomologica Scandinavica*. 42:1–2642007.

53. Stach, J. The Apterygotan Fauna of Poland in Relation to the World. Fauna of this Group of Insects, Family: Sminthuridae. Akademia Nauk. Polska.

54. Schneider C, Cruaud C, D'Haese CA. Unexpected diversity in Neelipleona revealed by molecular phylogeny approach (Hexapoda, Collembola). *Soil Organisms*. Senckenberg Museum für Naturkunde Görlitz; 83:383–982011.

55. Bridge PD, Roberts PJ, Spooner BM, Panchal G. On the Unreliability of Published DNA Sequences. *New Phytologist*. 2003; doi:10.1046/j.1469-8137.2003.00861.x.

56. Seah YG, Ariffin AF, Jaafar TNAM. Levels of COI divergence in Family Leiognathidae using sequences available in GenBank and BOLD Systems: A review on the accuracy of public databases. *Aquac Aquar Conserv Legis Int J Bioflux Soc*. 2017;10: 391–401.

 Click here to access/download;Table;Tables1_2.docx

Table 1. Species included in the phylogenetic analysis (taxonomic dataset expended from [44]).

Species	Order	Family	Repository	Accession	Source
Anopheles coluzzii	Diptera	Culicidae	NCBI	ASM413651v2	[3]
Catajapyx aquilonaris	Dicellurata	Japygidae	NCBI	GCA_000934665.2	[45]
Ceratophysella communis	Poduromorpha	Hypogastruridae	NCBI	GCA_009869905.1	[44]
Desoria tigrina	Entomobryomorpha	Isotomidae	EMBL-ENA	ERZ1473261	This study
Folsomia candida	Entomobryomorpha	Isotomidae	NCBI	GCA_002217175.1	[7]
Lipothrix lubbocki	Symphypleona	Sminthuridae	NCBI	GCA_009872335.1	[44]
Mesaphorura yosii	Poduromorpha	Tullbergiidae	NCBI	GCA_009869945.1	[44]
Neelides sp.	Neelipleona	Neelidae	NCBI	GCA_009869795.1	[44]
Oncopodura yosiiana	Entomobryomorpha	Oncopoduridae	NCBI	GCA_009869805.1	[44]
Orchesella cincta	Entomobryomorpha	Entomobryidae	NCBI	GCA_001718145.1	[43]
Pseudachorutes palmiensis	Poduromorpha	Neanuridae	NCBI	GCA_009869845.1	[44]
Pseudobourletiella spinata	Symphypleona	Bourletiellidae	NCBI	GCA_009870155.1	[44]
Pygmarrhopalites habei	Symphypleona	Arrhopalitidae	NCBI	GCA_009870185.1	[44]
Sinella curviseta	Entomobryomorpha	Entomobryidae	NCBI	GCA_004115045.1	[8]
Sminthurides aquaticus	Symphypleona	Sminthurididae	EMBL-ENA	ERZ1473260	This study
Sminthurides bifidus	Symphypleona	Sminthurididae	NCBI	GCA_009872375.1	[44]
Thalassaphorura encarpata	Poduromorpha	Onychiuridae	NCBI	GCA_009869925.1	[44]
Tomocerus qinae	Entomobryomorpha	Tomoceridae	NCBI	GCA_009869885.1	[44]

Table 2. Statistics of several assemblies generated from long read sequencing (with or without additional short reads) and/or low input approach.

Species	*Desoria tigrina*	*Sminthurides aquaticus*	*Folsomia candida*	*Orchesella cincta*	*Sinella curviseta*	*Anopheles coluzzii*	*Lycorma delicatula*
**Class**	**Collembola**	**Collembola**	Collembola	Collembola	Collembola	Insecta	Insecta
**Body size class**	**2 mm**	**1 mm**	2 mm	2 mm	2 mm	4 mm	20 mm
**N specimens in input**	**1 (PacBio)**	**1 (PacBio)**	1,600 (Pacbio) + 100 (Illumina)	40 (PacBio) + 1 (Illumina)	500 (PacBio) + 10 (Illumina)	1 (PacBio)	1 (PacBio)
**WGA**	**Yes**	**Yes**	No	No	No	No	No
**# contigs**	**142**	**79**	162	9,398	599	1,034	2,927
**Largest contig**	**14,592,742**	**19,603,089**	28,534,321	807,113	12,986,801	11,911,669	9,998,986
**Total length**	**211,462,971**	**165,915,169**	221,702,752	286,764,906	381,458,724	340,555,854	2,252,044,789
**N50**	**5,628,779**	**8,776,828**	6,519,406	65,879	3,284,409	2,625,112	1,519,606
**N75**	**2,264,114**	**4,641,270**	2,726,164	23,461	1,147,450	440,054	811,306
**L50**	**11**	**7**	8	925	32	36	434
**L75**	**28**	**13**	21	2,812	74	126	935
**Busco % C (D), F, M**	**95.8 (2.2), 1.2, 3.0**	**96.1 (1.6), 1.3, 2.6**	97.1 (0.9), 0.5, 2.4	94.5 (3.2), 1.8, 3.7	95.6, (4.4), 1.3, 3.1	99.6 (2.9), 0.0, 0.4	96.5 (1.9), 2.0, 1.5
**Publications**	**This study**	**This study**	[43]	[7]	[8]	[3]	[53]
**Assembly accession**	**EMBL-ENA: PRJEB39696**	**EMBL-ENA: PRJEB39696**	NCBI: ASM221717v1	NCBI:ASM171814v	NCBI:ASM411504v1	NCBI: ASM413651v2	DOI:10.15482/USDA.ADC/1503745

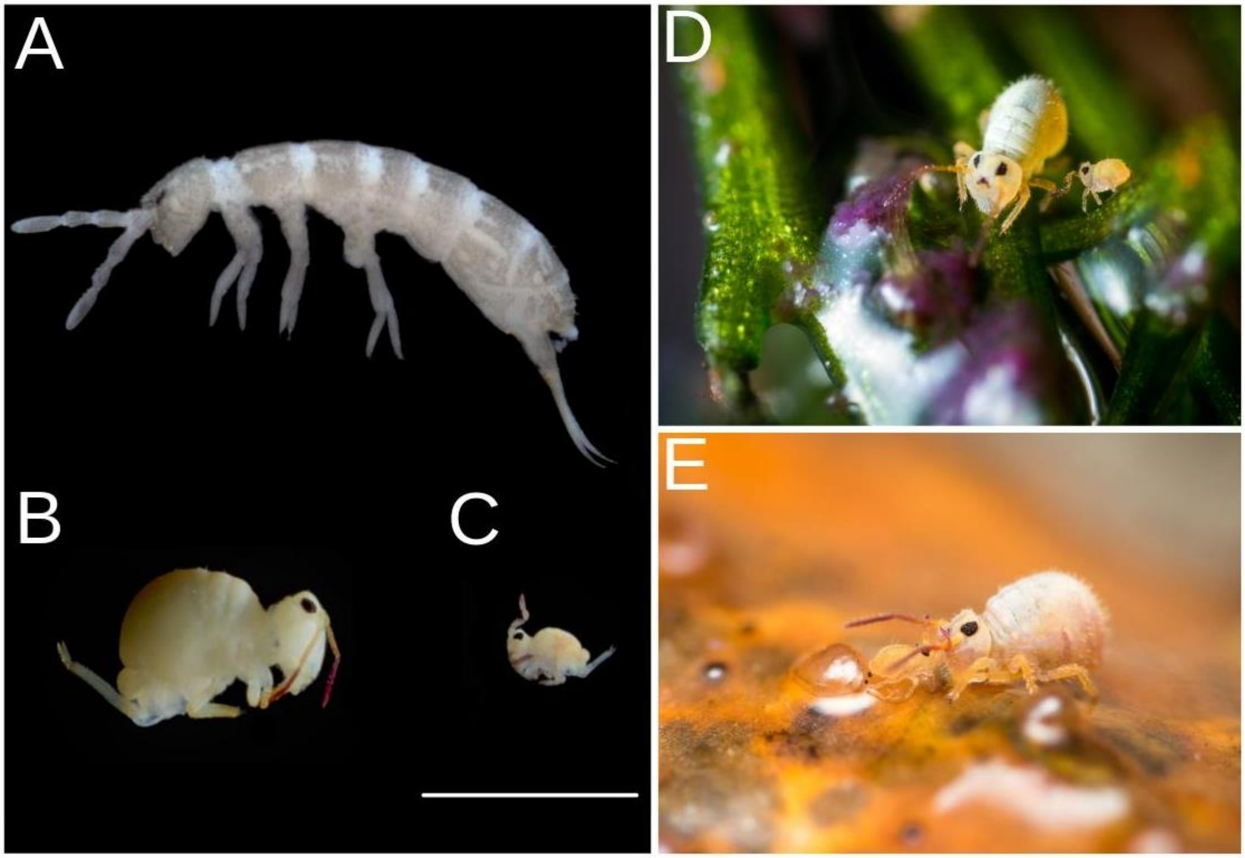

Figure 1. (A) *Desoria tigrina*. *Sminthurides aquaticus* (B) female, (C) male, (D) male and female on wet plant, (E) courtship on a floating dead twig: the male uses its clasping antennae to grab the antennae of the much bigger female. (A–C) Specimens preserved in 96% ethanol, scale bar = 1 mm.

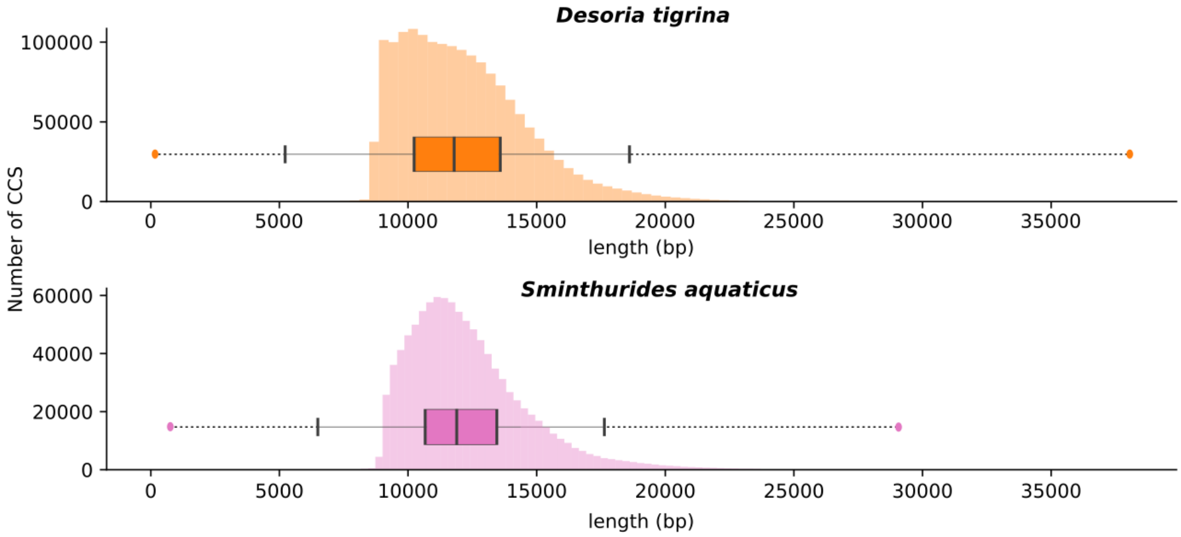

Figure 2. Distribution of CCS length. Outliers are not shown on the boxplot, except minimum and maximum length values each represented by a dot.

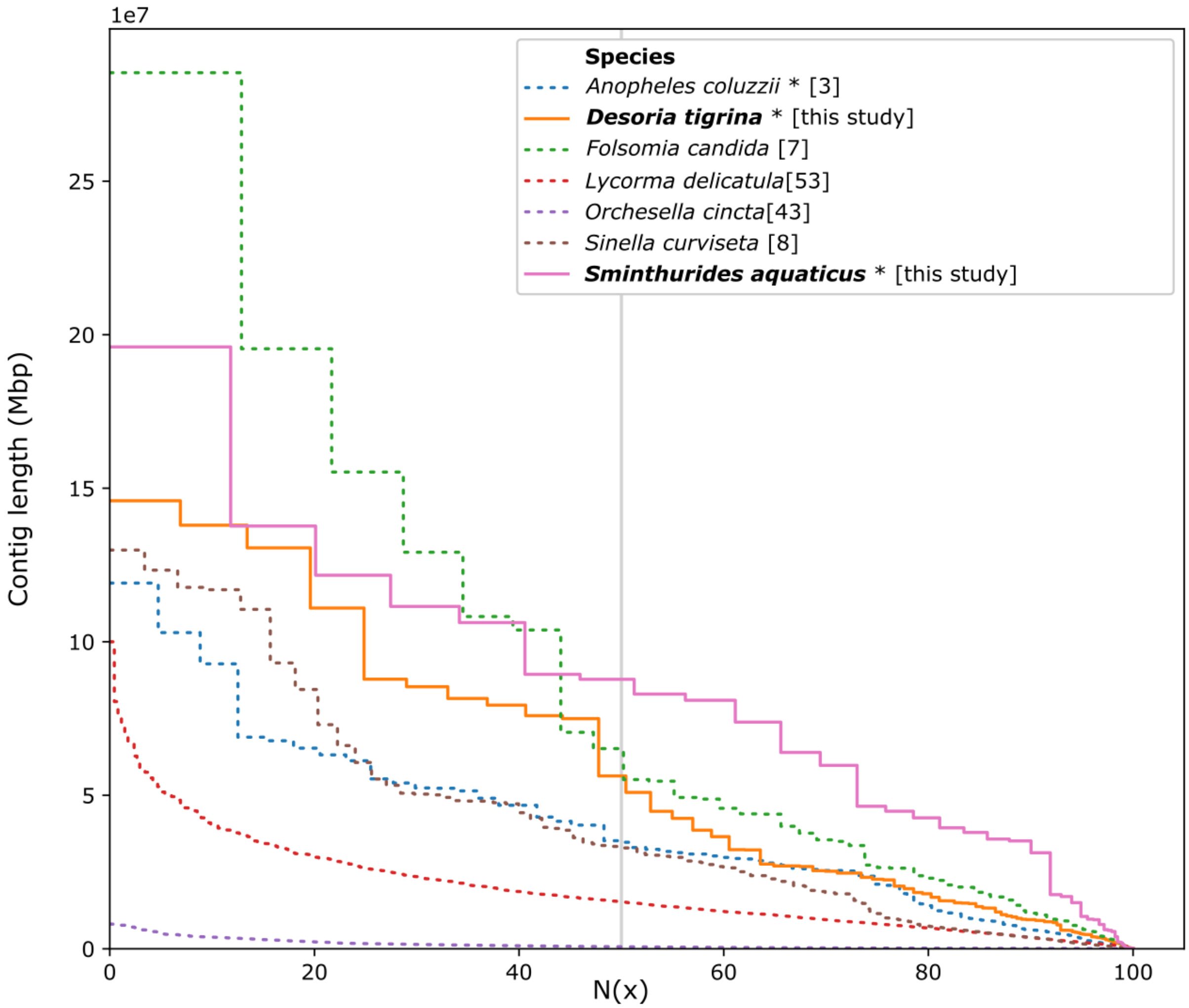

Figure 3. N(x) plot of recent high-quality genomes assembled with long reads, including the assemblies presented in this study.

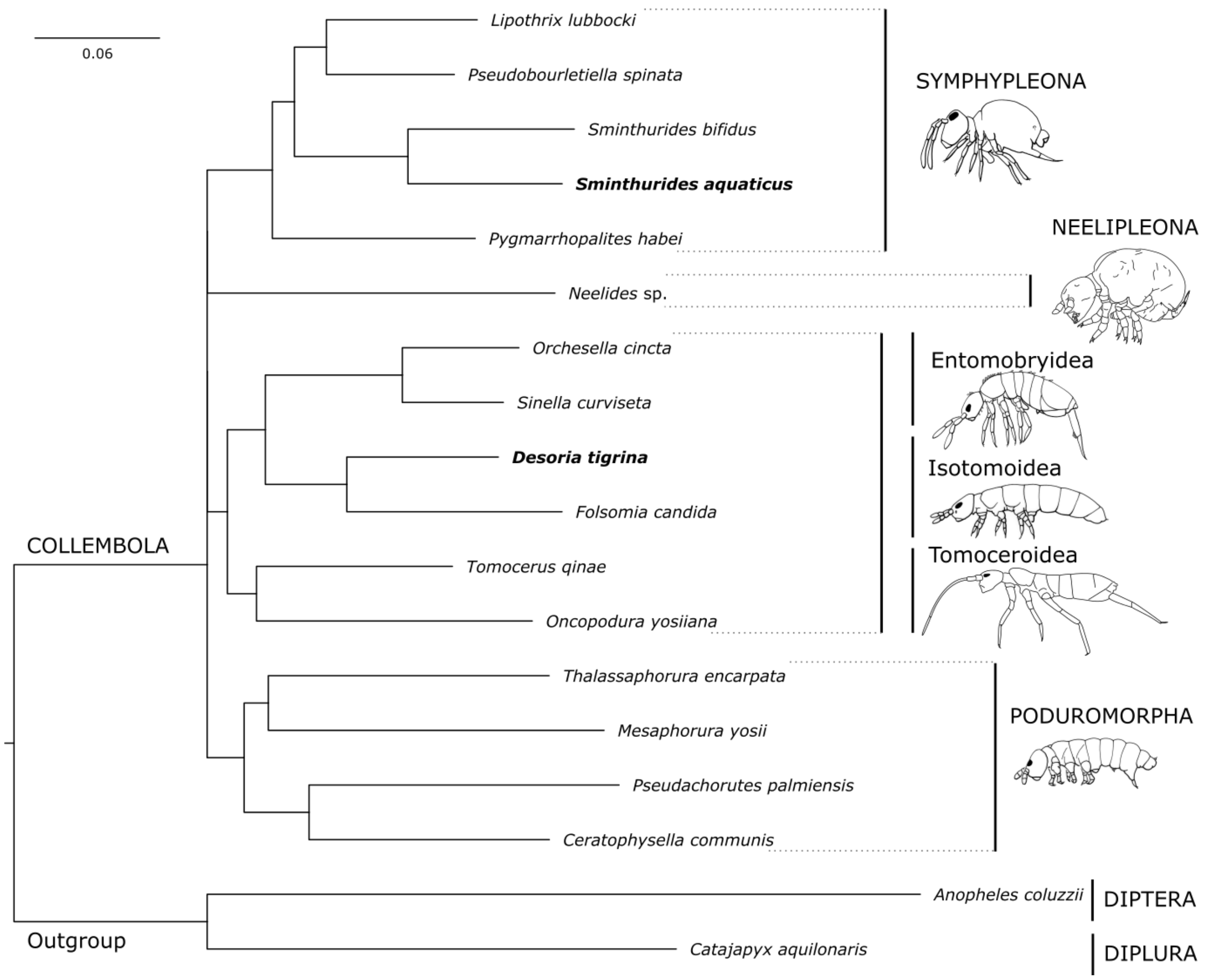

Figure 4. Phylogeny of Collembola based on the alignment of 545 protein sequences. Bootstrap support of shown nodes is 100%, nodes with bootstrap supports of 73 % were collapsed.

Click here to access/download
Supplementary Material
Supplementary_file_S1_FemtoPulse.pdf

Click here to access/download
Supplementary Material
S2_report_on_preliminary_assemblies.docx
