# Supplementary material for: Two high-quality de novo genomes from single ethanol-preserved specimens of tiny metazoans (Collembola)
Source: Gigascience. 2021 May 21;10(5):giab035. doi: 10.1093/gigascience/giab035 (PMC8138834; doi:10.1093/gigascience/giab035)
Supplement: giab035_Supplemental_Files [file giab035_supplemental_files.zip › S2_report_on_preliminary_assemblies.docx]

**S2: Report on preliminary assemblies.**

**Assembly statistics**

| **Species** | **Assembly tool** | **# contigs** | **Largest contig** | **Total length** | **GC (%)** | **N50** |
| --- | --- | --- | --- | --- | --- | --- |
| *Desoria tigrina* | Falcon curated^1^ | 453 | 7,399,450 | 210,360,062 | 37.2 | 1,032,224 |
| *Desoria tigrina* | Hifiasm | 309 | 13,849,643 | 232,521,345 | 37.35 | 3,752,039 |
| *Desoria tigrina* | Hifiasm and Purge_haplotigs^2^ | 160 | 13,835,527 | 220,675,542 | 37.31 | 3,752,039 |
| *Desoria tigrina* | wtdbg2 | 1,102 | 4,898,276 | 194,261,753 | 37.17 | 892,860 |
| *Desoria tigrina* | Flye | 5,139 | 2,703,854 | 335,822,651 | 37.23 | 145,370 |
| *Desoria tigrina* | HiCanu | 2,948 | 6,381,805 | 424,162,421 | 37.27 | 468,540 |
| *Desoria tigrina* | IPA | 707 | 7,110,412 | 330,032,605 | 37.19 | 769,387 |
| *Sminthurides aquaticus* | Falcon curated^1^ | 168 | 8,138,880 | 170,975,267 | 40.19 | 2,645,292 |
| *Sminthurides aquaticus* | Flye | 4,089 | 5,757,068 | 252,158,078 | 40.17 | 187,842 |
| *Sminthurides aquaticus* | HiCanu | 1,162 | 8,143,067 | 341,297,864 | 40.51 | 1,721,811 |
| *Sminthurides aquaticus* | HiCanu and Purge_haplotigs^2^ | 208 | 8,143,067 | 172,968,035 | 40.28 | 2,741,900 |
| *Sminthurides aquaticus* | Hifiasm | 292 | 19,618,543 | 184,801,495 | 41.15 | 8,304,850 |
| *Sminthurides aquaticus* | Hifiasm and Purge_haplotigs^2^ | 94 | 19,618,543 | 166,784,809 | 40.19 | 8,789,914 |
| *Sminthurides aquaticus* | IPA | 375 | 10,052,512 | 324,198,229 | 40.53 | 1,808,180 |
| *Sminthurides aquaticus* | IPA and Purge_haplotigs^2^ | 128 | 10,052,512 | 172,881,898 | 40.15 | 2,563,865 |
| *Sminthurides aquaticus* | wtdbg2 | 11 | 5,588,115 | 35,165,401 | 40.44 | 3,098,005 |

^1^See description for Falcon in the text below. ^2^Purge_haplotigs was applied in some instances to estimate the statistics of the haploid assembly, but it was not repeated for all preliminary assemblies.

**Assembly command lines.**

For Canu and wtdbg2 that required a genome size estimation as a parameter, we provided the following: *Desoria tigrina*: 220m, *Sminthurides aquaticus*: 170m.

**HiCanu from Canu** **version 2.1**

canu -d canu_run -p species_name genomeSize=###m -pacbio-hifi species_name_CCS.fasta

**Flye** **version 2.9.1-b1676**

flye -t 32 --pacbio-hifi species_name_CCS.fasta -o flye_run

**IPA version 1.1.2**

ipa local --nthreads 20 --njobs 4 -i species_name_CCS.fasta

**Hifiasm version** **0.12-r304**
hifiasm -o Saq.CCS.hifiasm.asm -t 24 species_name_CCS.fasta

**wtdbg2 version 2.5**

wtdbg2 -i species_name_CCS.fasta -o wtdbg2_run -t 16 -g ###m

wtpoa-cns -t 16 -i wtdbg2_run .ctg.lay.gz -fo wtdbg2_run .ctg.fa

**Falcon version** falcon-kit 1.8.0

Parameters adapted: genome_size = 200,000,000, -e.98 (ovlp_daligner_option), --min-idt 98 (overlap_filtering_setting)

^1^In the first draft of our project, we used Falcon and further curated the resulting assemblies for both species. Briefly this included haplotigs purging with purge_haplotigs for *S. aquaticus* and purge_dups for *D. tigrina*, assembly polishing with Racon, correction of mis-assemblies at some large NUMTs insertion point in the genomes, and removal of contigs from non-target organisms. Those steps were comparable to those presented in the final paper for the Hifiasm produced assemblies.
