# Supplementary material for: Two high-quality de novo genomes from single ethanol-preserved specimens of tiny metazoans (Collembola)
Source: Gigascience. 2021 May 21;10(5):giab035. doi: 10.1093/gigascience/giab035 (PMC8138834; doi:10.1093/gigascience/giab035)
Supplement: giab035_Supplemental_Files [file giab035_supplemental_files.zip › Supplementary_file_S1_FemtoPulse.pdf]

Sample: 4498.I.1_diluted_1:10 **Desoria tigrina**
Well Location: B1
Created: 22-Jan-20 3:59 PM

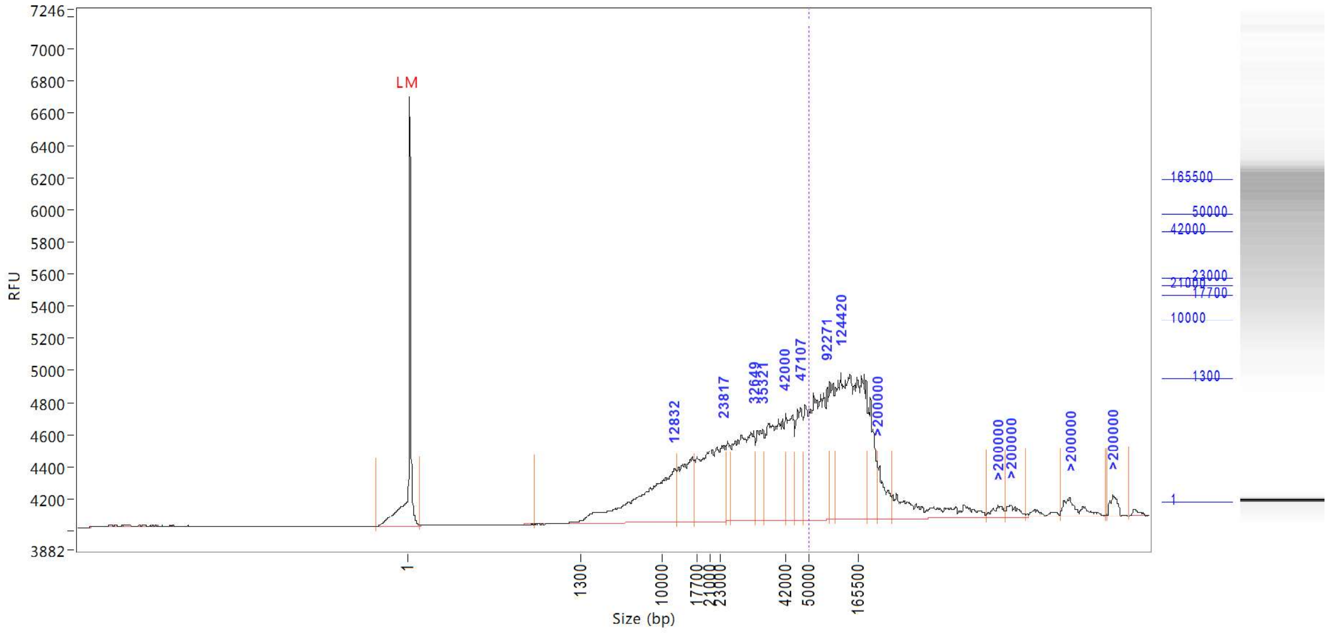

Peak	Size (bp)	Conc. (ng/uL)	From (bp)	To (bp)	Avg. Size (bp)	CV%	RFU	Corr. Peak Area
1	1 (LM)	0.0015	0	86	0	-182.99	2676	20.710
2	12832	0.0178	954	13284	8197	37.22	332	25.326
3	23817	0.0150	17417	24708	21156	9.56	478	21.336
4	32649	0.0133	25895	33168	29577	7.11	565	18.910
5	35321	0.0050	33168	35692	34428	2.15	566	7.098
6	42000	0.0128	35692	42001	38925	4.77	641	18.172
7	47107	0.0055	45235	48043	46640	1.76	703	7.824
8	92271	0.0176	48043	96439	68083	23.06	828	25.106
9	124420	0.0243	113109	188028	150100	14.39	915	34.583
10	>200000	0.0028	>200000	>200000	>200000	4.63	353	4.019
11	>200000	0.0007	>200000	>200000	>200000	2.53	74	1.058
12	>200000	0.0007	>200000	>200000	>200000	2.28	80	1.062
13	>200000	0.0015	>200000	>200000	>200000	3.64	119	2.116
14	>200000	0.0007	>200000	>200000	>200000	0.99	128	1.036

TIC:	0.1176	ng/uL
TIM:	0.0072	nmole/L
Total Conc.:	0.1481	ng/uL

GQN:	4.1
Threshold:	50000

```
Sample Peak Width (sec): 20   Sample Min Peak Height: 50    Sample Baseline V to V?: Y    Sample Baseline V to V pts: 3
Sample Filter: Binomial       # of Pts for Filter: 3        Sample Start Region (min): 0  Sample End Region (min): 70
Manual Baseline Start (min): 25     Manual Baseline End (min): 70
Marker Peak Width (sec): 8    Marker Min Peak Height: 500   Marker Baseline V to V?: N    Marker Baseline V to V pts: 3
Lower Marker Selection: First Peak > 500 RFU                Upper Marker Selection: Last Peak > 500 RFU
Ladder Size (bp)1, 1300, 10000, 17700, 21000, 23000, 42000, 50000, 165500
Quantification Using: Ladder          Final Concentration (ng/uL): 0.0200          Dilution Factor: 10.0
Min. RFU for Data Processing: 1       Size Threshold (b.p.): 50000
```


Copyright 2015 Advanced Analytical Technologies, Inc.

Sample: 4498.S.1_diluteted_1:4 *Sminthurides aquaticus*
Well Location: A7
Created: 24-Jan-20 1:42 PM

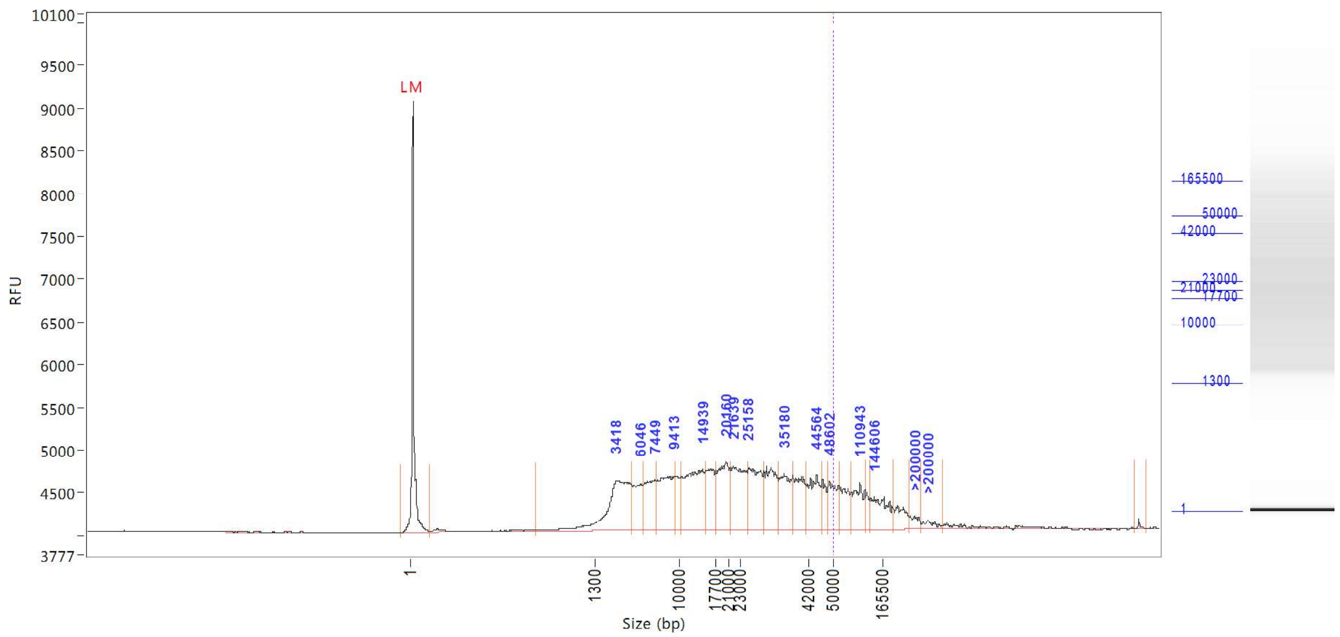

Peak	Size (bp)	Conc. (ng/uL)	From (bp)	To (bp)	Avg. Size (bp)	CV%	RFU	Corr. Peak Area
1	1 (LM)	0.0013	0	130	5	388.08	5054	35.897
2	3418	0.0116	884	5128	3382	35.07	591	31.177
3	6046	0.0040	5128	6250	5690	5.82	555	10.831
4	7449	0.0049	6250	7577	6918	5.63	599	13.239
5	9413	0.0077	7577	9592	8591	6.84	639	20.748
6	14939	0.0102	10266	15576	12953	11.91	709	27.231
7	20160	0.0066	17701	21256	19563	5.41	793	17.726
8	21639	0.0069	21256	24810	22789	4.44	751	18.385
9	25158	0.0062	24810	29404	27068	4.98	732	16.624
10	35180	0.0051	33440	37824	35611	3.60	657	13.742
11	44564	0.0043	41582	46195	43852	3.08	637	11.434
12	48602	0.0032	47981	63931	52680	9.34	573	8.597
13	110943	0.0031	88888	124292	106337	9.59	553	8.449
14	144606	0.0034	135319	189780	160723	9.74	381	8.997
15	>200000	0.0007	>200000	>200000	>200000	3.25	157	1.838
16	>200000	0.0006	>200000	>200000	>200000	4.89	115	1.675
17	0	0.0001	0	0	>200000	NaN	115	0.164

TIC:	0.0786	ng/uL
TIM:	0.3998	nmole/L
Total Conc.:	0.1010	ng/uL
GQN:	1.5	
Threshold:	50000	

Sample Peak Width (sec): 20 Sample Min Peak Height: 50 Sample Baseline V to V?: Y Sample Baseline V to V pts: 3
Sample Filter: Binomial # of Pts for Filter: 3 Sample Start Region (min): 0 Sample End Region (min): 70
Manual Baseline Start (min): 25 Manual Baseline End (min): 70
Marker Peak Width (sec): 8 Marker Min Peak Height: 500 Marker Baseline V to V?: N Marker Baseline V to V pts: 3
Lower Marker Selection: First Peak > 500 RFU Upper Marker Selection: Last Peak > 500 RFU
Ladder Size (bp)1, 1300, 10000, 17700, 21000, 23000, 42000, 50000, 165500
Quantification Using: Ladder Final Concentration (ng/uL): 0.0200 Dilution Factor: 10.0
Min. RFU for Data Processing: 1 Size Threshold (b.p.): 50000

 Copyright 2015 Advanced Analytical Technologies, Inc.
