# Supplementary material for: Two high-quality de novo genomes from single ethanol-preserved specimens of tiny metazoans (Collembola)
Source: Gigascience. 2021 May 21;10(5):giab035. doi: 10.1093/gigascience/giab035 (PMC8138834; doi:10.1093/gigascience/giab035)
Supplement: giab035_Response_to_Reviewer_Comments_Original_Submission [file giab035_response_to_reviewer_comments_original_submission.pdf]

Author's Response To Reviewer Comments

Close

Dear Dr. Hongling Zhou, dear reviewers

We submit the revision of our manuscript entitled “Two high-quality de novo genomes from single ethanol-preserved specimens of tiny metazoans (Collembola).” previously submitted as a Data Note to GigaScience.

We appreciated the comments of the reviewers, and took all the suggested corrections. Further typos were corrected in the text. The changes were recorded using the tracking modification tools of Microsoft office. They can all be accepted. We added the flowchart requested by reviewer #1.
Furthermore, we wish to deposit the alternative haplotig assembly of both species in GigaDB, as additional supporting data, as reviewer #2 inquired about them.

We give our detailed answers to the questions and suggestions of the reviewers below.

Response to reviewers:

>Reviewer #1 (Dr. Arong Lu): First, I'd like to commend the authors on attempting to sequence whole genomes of tiny metazoans, which account for a large part of biodiversity in nature and yet are difficult to be sequenced. Second, I am impressed by their ethanol-preserved specimens, which thus make genome sequencing more applicable and attractive in practice. We must admit that sometimes we cannot use fresh specimens directly for genome sequencing. Thus, I think this manuscript is really of scientific significance for specific fields such as insects.
>I found that the focal part of their sequencing protocol is the "whole genome amplification-based Ultra-Low DNA Input Workflow for SMRT Sequencing (PacBio)" throughout the text, which of course is very complex. So, I suggest the authors provide a flowchart showing critical or main steps during their workflow, and the readers can then understand easily and refer to their workflow in future projects.
We agreed with Reviewer #1 that a flowchart would help the readers to implement the workflow in their projects and thus added it to the manuscript (new Figure 2, figures numbering shifted accordingly).
>Finer points:
>Line 35: I suggest providing specific/important information for the 'novel' protocol herein.
We added some information about the ultra protocol in the abstract, as suggested.
>Line119-120: Are the specimens later for DNA extraction also morphologically identified?
The sequenced specimens were crushed and ground, therefore morphological identification was limited to stereomicroscope observations. Accurate identifications were done on many specimens collected together with the sequenced specimens. We slightly modified the paragraph to clarify our approach.
>Line130-131: The DNA extract was selected randomly or based on certain measurements?
DNA extract were selected randomly, we added this precision to the text.

>Reviewer #2 (Dr. Mahul Chakraborty): In "Two high-quality de novo genomes from single ethanol-preserved specimens of tiny metazoans (Collembola)." Schneider et al. described de novo genome assemblies of two tiny field collected Collembolan specimens. The authors collected high quality genomic DNA from the specimens following a Pacfiic Biosciences recommended protocol for ultra low input library, amplified them, and generated adequate sequence coverage to generate contiguous assemblies. This is a significant step forward in generating de novo genome assemblies from small amounts of tissues and cells and therefore will be a useful guide for not only people who are studying whole organisms but also people who are studying variation between cell or tissue types within an individual.
>I have some minor comments:
>"They were preserved in 96% ethanol, kept at ambient-temperature for one day until they would be stored at -20°C for 1.5 months, until DNA extraction."
>Was the preservation at -20 a deliberate step to see the effect of this treatment on sequencing or just a conscious choice for specimen preservation?
It was a conscious choice for specimen preservation. Cold storage in ethanol is a common practice to preserve specimens for future sequencing. When the possibility to sequence S. aquaticus came, we tried

with this relatively “fresh” sample and were happy with the results. We modified slightly the paragraph to better reflect that the -20°C was a storage step, and not an experiment.
>The specific conditions used (e.g. the time and speed of centrifuge) for the g-Tube shearing needs to be added in the Methods.
We added them.
>"Circularity was validated manually, and nucleotide bases were called with a 75% threshold Consensus.?" - please clarify what the 75% threshold consensus is.
We mean 75% majority-rule consensus, i.e. a base is called when in agreement with at least 75% of the covering reads. In practice, all bases were resolved without ambiguity. We corrected the text to clarify.
>"We then performed another estimation of the genome size by dividing the number of mapped nucleotides by mode of the coverage distribution" - Why was this done? Did the authors suspect the Genomescope estimate to be incorrect?
Both methods were proposed to estimate the genome size. We were curious to see if the following values genomescope estimate, assembly size and genome size estimation from mapped reads would remain close to each other.
>"We compared our new genomes sequenced to previous Collembola assemblies that were generated with long read and sometimes additional short read data." - This statement needs citations for the previous Collembola assemblies.
This is right, we added the references that belongs with the sentence. Those references were already cited in other part of the text.
>The authors used blastn and megablast to search the beta-lactams synthesis genes in the new assembly. Tblastx might be more appropriate.
We repeated the search with tblastn to query the protein sequences; and also repeated the search on the alternate haplotype sequences. Results are not changed. We added the precision to the text.
>"For D. tigrina a total of 20,22 Gb HiFi data (Q>=20) was generated," - Do you mean 20.22 ?
Yes, this is a typo.
>"For S. aquaticus a total of Gb HiFi data (Q>=20) was generated" - missing the number before Gb
We apologize for the typo, and added the value to the text: 12.4 Gb.
>The authors report only one assembly from hifiasm, which I presume is the primary assembly. Given that the authors assembled diploid individuals, I am curious whether hifiasm assembled the alternate haplotype sequences.
Following Reviewer #2 suggestion, we now report the alternative haplotig assembly. We took the alternative haplotig produced by hifiasm and concatenated them to the haplotigs purged from the primary assembly. We applied Purge_dups and our decontamination strategy to get a clean alternative haplotig assembly. We performed a BUSCO search on the alternative assembly and found >87% complete BUSCOs for each species. We now report the alternative assembly in the manuscript, and deposit them in the Giga-DB, as supporting material.
>"The insect genomes have higher BUSCO scores (96.5 and 99.6%), but lower contiguity (Table 2, Fig. 3)." - This statement is incorrect. A number of insect genomes are more contiguous than the assemblies presented here, including Drosophila melanogaster (PMID: 31653862) and several other Drosophila species, Anopheles stephensi (DOI:10.1101/2020.05.24.113019), Anopheles albimanus (PMID: 32883756)
Our statement was ambiguous, and thus maybe misunderstood. We compared our assembly to what we estimated being relevant examples, namely insect genomes previously sequenced using a single specimen with a low-input DNA approach, and assembled only with PacBio long reads (no Hi-C scaffolding). Naturally, chromosome level assemblies are available for several insect model species such as fruit-fly; but this is not a useful comparison. For example, in “DOI:10.1101/2020.05.24.11301”, the species is sequenced from 70 inbred specimens. The purpose of the limited comparison we offer in the manuscript is to report similar or improved results from 1- previously genomes of Collembola (more similar organism to our species, but sequenced from large pool of fresh specimens where we worked with single ethanol preserved specimens); 2- previously low input PacBio sequenced insects (still bigger animals than our Collembola, and fresh). We edited the text to disambiguate the statement.

We are happy to acknowledge the help of the reviewers, Dr. Arong Luo and Dr. Mahul Chakraborty, in reviewing our work; and thank them for their suggestions and corrections that improved the quality of the manuscript.
We hope to see our manuscript accepted for publication in Gigascience. As soon as we get a validation, we will request the public release of the data in ENA-EMBL and provide the definitive accession numbers for those data.

On behalf of the authors,
Dr. Clément Schneider

Sektionsleiter Apterygota
Senckenberg Museum für Naturkunde Görlitz
PF 30 01 54
02806 Görlitz
Tel.: +49 3581 4760-551

Close
