# Supplementary material for: Two high-quality de novo genomes from single ethanol-preserved specimens of tiny metazoans (Collembola)
Source: Gigascience. 2021 May 21;10(5):giab035. doi: 10.1093/gigascience/giab035 (PMC8138834; doi:10.1093/gigascience/giab035)
Supplement: giab035_Reviewer_1_Report_Original_Submission — Arong Luo -- 1/9/2021 Reviewed [file giab035_reviewer_1_report_original_submission.pdf]

Reviewer Report

Title: Two high-quality de novo genomes from single ethanol-preserved specimens of tiny metazoans (Collembola).

Version: Original Submission **Date:** 1/9/2021

Reviewer name: Arong Luo

Reviewer Comments to Author:

First, I'd like to commend the authors on attempting to sequence whole genomes of tiny metazoans, which account for a large part of biodiversity in nature and yet are difficult to be sequenced. Second, I am impressed by their ethanol-preserved specimens, which thus make genome sequencing more applicable and attractive in practice. We must admit that sometimes we cannot use fresh specimens directly for genome sequencing. Thus, I think this manuscript is really of scientific significance for specific fields such as insects.
I found that the focal part of their sequencing protocol is the "whole genome amplification-based Ultra-Low DNA Input Workflow for SMRT Sequencing (PacBio)" throughout the text, which of course is very complex. So, I suggest the authors provide a flowchart showing critical or main steps during their workflow, and the readers can then understand easily and refer to their workflow in future projects.
Finer points:
Line 35: I suggest providing specific/important information for the 'novel' protocol herein.
Line119-120: Are the specimens later for DNA extraction also morphologically identified?
Line130-131: The DNA extract was selected randomly or based on certain measurements?
Line 393: delete the dot '.'

Level of Interest

Please indicate how interesting you found the manuscript: Choose an item.

Quality of Written English

Please indicate the quality of language in the manuscript: Choose an item.

Declaration of Competing Interests

Please complete a declaration of competing interests, considering the following questions:

- Have you in the past five years received reimbursements, fees, funding, or salary from an organisation that may in any way gain or lose financially from the publication of this manuscript, either now or in the future?

- Do you hold any stocks or shares in an organisation that may in any way gain or lose financially from the publication of this manuscript, either now or in the future?
- Do you hold or are you currently applying for any patents relating to the content of the manuscript?
- Have you received reimbursements, fees, funding, or salary from an organization that holds or has applied for patents relating to the content of the manuscript?
- Do you have any other financial competing interests?
- Do you have any non-financial competing interests in relation to this paper?

If you can answer no to all of the above, write 'I declare that I have no competing interests' below. If your reply is yes to any, please give details below.

I declare that I have no competing interests.

I agree to the open peer review policy of the journal. I understand that my name will be included on my report to the authors and, if the manuscript is accepted for publication, my named report including any attachments I upload will be posted on the website along with the authors' responses. I agree for my report to be made available under an Open Access Creative Commons CC-BY license (http://creativecommons.org/licenses/by/4.0/). I understand that any comments which I do not wish to be included in my named report can be included as confidential comments to the editors, which will not be published.

Choose an item.

To further support our reviewers, we have joined with Publons, where you can gain additional credit to further highlight your hard work (see: https://publons.com/journal/530/gigascience). On publication of this paper, your review will be automatically added to Publons, you can then choose whether or not to claim your Publons credit. I understand this statement.

Yes Choose an item.
