# Supplementary material for: Two high-quality de novo genomes from single ethanol-preserved specimens of tiny metazoans (Collembola)
Source: Gigascience. 2021 May 21;10(5):giab035. doi: 10.1093/gigascience/giab035 (PMC8138834; doi:10.1093/gigascience/giab035)
Supplement: giab035_Reviewer_2_Report_Original_Submission — Mahul Chakraborty -- 1/19/2021 Reviewed [file giab035_reviewer_2_report_original_submission.pdf]

Reviewer Report

Title: Two high-quality de novo genomes from single ethanol-preserved specimens of tiny metazoans (Collembola).

Version: Original Submission **Date:** 1/19/2021

Reviewer name: Mahul Chakraborty

Reviewer Comments to Author:

In "Two high-quality de novo genomes from single ethanol-preserved specimens of tiny metazoans (Collembola)." Schneider et al. described de novo genome assemblies of two tiny field collected Collembolan specimens. The authors collected high quality genomic DNA from the specimens following a Pacfiic Biosciences recommended protocol for ultra low input library, amplified them, and generated adequate sequence coverage to generate contiguous assemblies. This is a significant step forward in generating de novo genome assemblies from small amounts of tissues and cells and therefore will be a useful guide for not only people who are studying whole organisms but also people who are studying variation between cell or tissue types within an individual.
I have some minor comments:
"They were preserved in 96% ethanol, kept at ambient-temperature for one day until they would be stored at -20Â°C for 1.5 months, until DNA extraction." -
Was the preservation at -20 a deliberate step to see the effect of this treatment on sequencing or just a conscious choice for specimen preservation?
The specific conditions used (e.g. the time and speed of centrifuge) for the g-Tube shearing needs to be added in the Methods.
"Circularity was validated manually, and nucleotide bases were called with a 75% threshold Consensus.?" - please clarify what the 75% threshold consensus is.
"We then performed another estimation of the genome size by dividing the number of mapped nucleotides by mode of the coverage distribution" - Why was this done? Did the authors suspect the Genomescope estimate to be incorrect?
"We compared our new genomes sequenced to previous Collembola assemblies that were generated with long read and sometimes additional short read data." - This statement needs citations for the previous Collembola assemblies.
The authors used blastn and megablast to search the beta-lactams synthesis genes in the new assembly. Tblastx might be more appropriate.
"For D. tigrina a total of 20,22 Gb HiFi data (Q>=20) was generated," - Do you mean 20.22 ?
"For S. aquaticus a total of Gb HiFi data (Q>=20) was generated" - missing the number before Gb
The authors report only one assembly from hifiasm, which I presume is the primary assembly. Given that the authors assembled diploid individuals, I am curious whether hifiasm assembled the alternate haplotype sequences.
"The insect genomes have higher BUSCO scores (96.5 and 99.6%), but lower contiguity (Table 2, Fig. 3)." - This statement is incorrect. A number of insect genomes are more contiguous than the assemblies

presented here, including Drosophila melanogaster (PMID: 31653862) and several other Drosophila species, Anopheles stephensi (DOI:10.1101/2020.05.24.113019), Anopheles albimanus (PMID: 32883756)

Level of Interest

Please indicate how interesting you found the manuscript: Choose an item.

Quality of Written English

Please indicate the quality of language in the manuscript: Choose an item.

Declaration of Competing Interests

Please complete a declaration of competing interests, considering the following questions:

- Have you in the past five years received reimbursements, fees, funding, or salary from an organisation that may in any way gain or lose financially from the publication of this manuscript, either now or in the future?
- Do you hold any stocks or shares in an organisation that may in any way gain or lose financially from the publication of this manuscript, either now or in the future?
- Do you hold or are you currently applying for any patents relating to the content of the manuscript?
- Have you received reimbursements, fees, funding, or salary from an organization that holds or has applied for patents relating to the content of the manuscript?
- Do you have any other financial competing interests?
- Do you have any non-financial competing interests in relation to this paper?

If you can answer no to all of the above, write 'I declare that I have no competing interests' below. If your reply is yes to any, please give details below.

I declare that I have no competing interests

I agree to the open peer review policy of the journal. I understand that my name will be included on my report to the authors and, if the manuscript is accepted for publication, my named report including any attachments I upload will be posted on the website along with the authors' responses. I agree for my report to be made available under an Open Access Creative Commons CC-BY license (http://creativecommons.org/licenses/by/4.0/). I understand that any comments which I do not wish to be included in my named report can be included as confidential comments to the editors, which will not be published.

Choose an item.

To further support our reviewers, we have joined with Publons, where you can gain additional credit to further highlight your hard work (see: https://publons.com/journal/530/gigascience). On publication of this paper, your review will be automatically added to Publons, you can then choose whether or not to claim your Publons credit. I understand this statement.

Yes Choose an item.
